# Beyond Trinucleotide Repeat Expansion in Fragile X Syndrome: Rare Coding and Noncoding Variants in *FMR1* and Associated Phenotypes

**DOI:** 10.3390/genes12111669

**Published:** 2021-10-22

**Authors:** Cedrik Tekendo-Ngongang, Angela Grochowsky, Benjamin D. Solomon, Sho T. Yano

**Affiliations:** 1 National Human Genome Research Institute, National Institutes of Health, Bethesda, MD 20892, USA; cedrik.ngongang@nih.gov (C.T.-N.); solomonb@mail.nih.gov (B.D.S.); 2 Department of Pediatrics, Vanderbilt University Medical Center, Nashville, TN 37232, USA; angela.grochowsky@vumc.org

**Keywords:** *FMR1*, FMRP, fragile X, variant classification, copy number variants

## Abstract

*FMR1* (FMRP translational regulator 1) variants other than repeat expansion are known to cause disease phenotypes but can be overlooked if they are not accounted for in genetic testing strategies. We collected and reanalyzed the evidence for pathogenicity of *FMR1* coding, noncoding, and copy number variants published to date. There is a spectrum of disease-causing *FMR1* variation, with clinical and functional evidence supporting pathogenicity of five splicing, five missense, one in-frame deletion, one nonsense, and four frameshift variants. In addition, *FMR1* deletions occur in both mosaic full mutation patients and as constitutional pathogenic alleles. De novo deletions arise not only from full mutation alleles but also alleles with normal-sized CGG repeats in several patients, suggesting that the CGG repeat region may be prone to genomic instability even in the absence of repeat expansion. We conclude that clinical tests for potentially *FMR1*-related indications such as intellectual disability should include methods capable of detecting small coding, noncoding, and copy number variants.

## 1. Introduction

Fragile X syndrome (FXS) is an X-linked disorder due to a loss of *FMR1* function. The phenotype involves both neurological and physical features, including developmental delays (DD), intellectual disability (ID), autism spectrum disorder (ASD), attention-deficit/hyperactivity disorder (ADHD), joint hypermobility, and characteristic facial features such as a long face, large or prominent ears, prominent forehead, and prominent jaw [1,2]. Because not all features are present in all affected individuals, *FMR1* pathogenic variants can be found (or may not be recognized) in patients with apparently nonsyndromic ID or ASD. FXS phenotypes are not restricted to hemizygous males; females heterozygous for loss-of-function variants can also be clinically affected [2,3].

Expansions of the CGG repeat located in the *FMR1* 5′ untranslated region (UTR) account for most cases of *FMR1-*related disease through both loss- and gain-of-function mechanisms. The “normal” repeat size is 5–44 repeats [4]. “Full mutation” expansions to >200 CGG repeats typically result in gene silencing with methylation of the *FMR1* promoter; this loss-of-function mechanism is responsible for most cases of FXS [4]. “Premutations” containing 55–200 repeats confer risk for expansion to full mutations in the next generation but can also themselves cause non-FXS phenotypes due to toxicity of RNA expressed from the premutation allele, which is not silenced and can even display increased levels of transcription [5]. These include fragile X-associated tremor/ataxia syndrome, fragile X-associated primary ovarian insufficiency, and neurodevelopmental and psychiatric conditions. The same gain-of-function mechanism and phenotypes can also occur in individuals with unmethylated full mutation alleles that are transcribed [5]. “Intermediate” alleles ranging from 45 to 54 repeats display a higher risk for repeat length change in the next generation but not an expansion to full mutation within one generation.

Most clinical tests and guidelines to date have thus focused on detecting CGG repeat expansions [4]. Some but not all clinical tests for repeat expansion can also detect interspersion of AGG repeats, which stabilize the repeat region and reduce the probability of its expansion [6,7]. However, the common strategy of ordering CGG repeat expansion testing “for *FMR1*” means that a negative result does not eliminate *FMR1*-related disorders from the differential diagnosis. Besides random mutations, the *FMR1* region is prone to genomic instability since long tracts of CGG repeats are mutagenic on nearby sequences; they fold into non-B-DNA conformations and produce double-stranded DNA breaks from replication fork stalling [8,9]. It is thus important to consider the possibility of loss-of-function *FMR1* variants other than repeat expansions when ordering, designing, or interpreting clinical tests for potentially *FMR1*-related phenotypes such as ID and ASD.

To better understand the range of pathogenic variants in *FMR1* other than repeat expansions, we collected previously reported variants other than CGG repeat length changes and analyzed their evidence for pathogenicity. We included the entire range of variation with coding, noncoding, and copy number variants and provided a curated list of evidence relevant to the impact of each variant to assist with independent reassessment of pathogenicity. We further analyzed the inheritance and allelic origin of deletions to understand whether rearrangements originate from repeat expansion alleles.

## 2. Materials and Methods

All previously reported *FMR1* variants other than CGG repeat length changes were collected from PubMed search results for the terms (*FMR1*) OR (“fragile X”) through August 2021 (8572 results) by manually identifying publications with *FMR1* variants identified in humans, followed by filtering for relevant variants as detailed below. Variants were mapped to the GRCh38.p13 chromosome X (NC_000023.11) and *FMR1* transcript NM_002024.6 reference sequences. Coordinates from previous assemblies were converted using the UCSC Genome Browser and LiftOver (http://genome.ucsc.edu (accessed on 1 August 2021)) [10]. On these reference sequences, the transcription start site is c.-261 (g.147911919), and the CGG repeat is c.-129 to c.-70 (g.147912051–147912110).

The pathogenicity of collected *FMR1* variants was reclassified by board-certified medical geneticists following the American College of Medical Genetics and Genomics/Association for Molecular Pathology (ACMG/AMP) joint guidelines for sequence variant interpretation (SVI) [11] and ACMG/ClinGen technical standards for constitutional copy number variant (CNV) interpretation [12]. For the SVI criteria, since FMRP has multiple functions and a single standardized functional test does not exist, the PS3 criterion was applied for functional studies showing a deleterious effect on any physiologically relevant function. The BS2 criterion was applied for noncoding/splice variants with putative effects on protein expression given that loss of FMRP expression is fully penetrant in males, but not variants expected to change protein sequence since the penetrance of missense variants in *FMR1* has not been established.

For CNVs, all variants that met the inclusion criteria necessarily fell under criteria 1A (contains protein-coding gene) and 3A (<35 protein-coding genes involved) on both the copy number loss and gain scoring metrics; therefore, these criteria are not shown. Section 4 was also inapplicable to the deletion variants, given that *FMR1* loss is known to cause disease and is not shown. Criterion 5A, which is subject to interpreter discretion, was assigned 0.15 points for all reported de novo occurrences due to the moderate genetic heterogeneity of fragile X-like phenotypes and since confirmation of maternity was not always explicitly specified.

Interpretation of partial deletions can also vary considerably between different raters based on how the guidelines are interpreted. For consistency, all such deletions involving coding sequence were scored as intragenic variants following the SVI guidelines with ClinGen recommendations for modifying PVS1, as recommended in the CNV interpretation technical standard [12,13]. The edge case of deletion extending from upstream sequences into part of the FMRP N-terminus within exon 1 was interpreted as PVS1_Moderate regardless of the location of the 5′ breakpoint. Criterion PS2 was applied to all de novo variants, though an argument could be made for assigning a decreased weight when the originating allele is a full mutation expansion. Criterion PM2 was also applied due to the absence of similar coding region deletions in the Database of Genomic Variants; though this approach has limitations, it is often used [14].

On the other hand, all such deletions that ended upstream to c.1, to which most of the SVI criteria are not applicable, were interpreted as 2C-2 under the CNV interpretation scoring metric. The recommendation to upgrade points for deletions involving well-characterized promoter regions was interpreted as a score of 0.30 for large deletions including the promoter and 0 for 5′UTR deletions within exon 1. This left two CNVs with additional benign evidence (normal *FMR1* expression in proband) that are noted in Table 1.

Both small variants and copy number variants were included as follows: both coding and noncoding small variants were included if reported in association with a phenotype, while variants found among large sequencing cohorts were only included if absent in the Genome Aggregation Database (gnomAD v3.1.10; https://gnomad.broadinstitute.org (accessed on 1 August 2021)) or specifically discussed in the literature. For CNVs, deletions that extended into the CGG repeat region were included, while deletions entirely within the CGG repeat (i.e., repeat contractions) were out of the scope of this paper. To focus on *FMR1*-related phenotypes, we excluded larger variants such as cytogenetically visible microdeletions, other CNVs affecting neighboring genes with known phenotypes (e.g., *AFF2* (FRAXE) and *IDS* (mucopolysaccharidosis II) distally) and X-autosome translocations.

We determined or estimated breakpoints of CNVs that were published without specific genomic coordinates as follows. If junction sequences were published, they were matched to the reference genome by NCBI BLAST (https://blast.ncbi.nlm.nih.gov/Blast.cgi (accessed on 1 August 2021)); breakpoints were shifted downstream of their published locations if the 3′ rule applied. If breakpoint locations were specified in reference to landmarks (e.g., c.1, the CGG repeat, or the other breakpoint) or to sequences such as pE5.1 (Genbank X61378), an approximate value was calculated using the appropriate sequence. If sequence-tagged site markers were specified and primer sequences for those markers were available in the NCBI Probe legacy database (UNISTS_human.sts file at https://ftp.ncbi.nih.gov/pub/ProbeDB/ (accessed on 1 August 2021)), the location of the primer sequences were determined by BLAST and checked against the STS track of the UCSC Genome Browser (https://genome.ucsc.edu/cgi-bin/hgGateway (accessed on 1 August 2021)). For site DXS296 (probe VK21C), while we were not able to find published primer sequences, it is distal to FRAXE and within the *AFF2* transcript [42]. If breakpoints were determined by restriction fragment length polymorphism (RFLP), no re-analysis was performed to avoid possible misidentification due to ambiguity in some of the restriction fragment sizes.

Conservation of protein residues involved in missense variants was visualized using ClustalOmega [43] to align FMR1P, FXR1P, and FXR2P protein sequences (Uniprot Q06787, P51114, P51116) and ConSurf [44].

## 3. Results

### 3.1. FMR1 CNVs in the Absence of Repeat Expansion

The majority of reported *FMR1* CNVs were deletions identified in patients who underwent clinical testing for neurological features such as DD, ID, ASD, and/or epilepsy, with four small duplications only containing *FMR1* (Table 1). Details of the clinical evidence used to assign pathogenicity criteria, and the phenotypes are listed in Supplementary Tables S1 and S2. In summary, pathogenic deletions involving the whole gene or eliminating c.1 were found in both male and heterozygous female probands, with presentations ranging from typical FXS to nonsyndromic epilepsy. On the other hand, most deletions within the promoter/5′UTR remained variants of uncertain significance (VUS) if the CNV interpretation criteria were strictly applied. Four deletion alleles were also found in individuals who were tested for reasons other than the presence of clinical manifestations. Two were in heterozygous females ascertained through a population screening study of pregnant women [33]. Two were in individuals hemizygous for de novo 5′UTR deletions tested due to family history of FXS, a 19-month-old male with c.-156_-69del and no neurological or physical findings, and a 10-year-old female with c.-196_-40del in trans with a large deletion including *FMR1* but no typical phenotype for FXS (only features were low birthweight, early thelarche, hearing loss, perinatal asphyxia with 6-week ICU stay) [34,35].

Proximal breakpoints were generally more frequent near the *FMR1* transcription start site/exon 1, rather than being evenly distributed along the length of the intergenic region upstream of *FMR1*. Breakpoints clustered near a previously described chi-like sequence as well as within long and short interspersed nuclear elements (LINEs, SINEs) and at regions of a few base pairs of microhomology (Table 1) [18].

Deletions were observed to occur on both full mutation and normal-sized CGG repeat alleles. Among eight de novo constitutional deletions for which maternal CGG repeat status was known, three originated from normal-sized alleles (one confirmed by haplotype to be from a 19-repeat allele), three originated from full mutation alleles by haplotype analysis, and two occurred in probands whose mothers were full mutation heterozygotes. Inheritance of deletion alleles from heterozygous and mosaic mothers was also observed, including cases of maternal germline mosaicism with recurrence in subsequent pregnancies despite negative maternal testing [20,23].

### 3.2. Mosaicism for CNVs with CGG Repeat Expansions

Repeat length instability is well-established in patients with expanded alleles, with somatic mosaicism for premutation to full mutation-sized repeats as well as repeat contractions. However, repeat expansions were also associated with mosaicism for CNVs around the repeat region. We did not apply clinical classification criteria to variants co-occurring with repeat expansions since the majority of cells in these individuals carried known pathogenic full mutation expansions.

All such CNVs reported were deletions, mostly starting proximal to the CGG repeat region (Table 2). While several breakpoints were observed multiple times in unrelated individuals, there was no single recurrent location. Furthermore, while some breakpoints had a few bases of microhomology [45,46,47], others were reported as lacking sequence features that might explain the breakpoint.

### 3.3. Coding Region Variants

Missense, in-frame deletion, frameshift, and nonsense variants have all been reported in patients with neurological and/or physical features of *FMR1*-associated disease (Table 3, Supplementary Table S3). A total of 11 published variants from all of these categories had sufficient evidence to conclude pathogenicity. We reclassified one variant that was reported as pathogenic, as well as three others reported as possibly causing disease, as VUSs under strict application of variant interpretation criteria (Table 3). In particular, c.1550C>T (p.P517L) was reported as a pathogenic stop-gain variant (p.Q406*) in a man with seizures and unilateral cerebral white matter hyperintensity [63]. This variant produces a stop codon in an alternate C-terminus in a different reading frame from that of the canonical isoform, such that we would have interpreted it as a missense VUS rather than loss-of-function based on the updated recommendations for applying the PVS1 criterion [13].

An expanded analysis of all published variants with the clinical evidence used to assign pathogenicity criteria, including variants from sequencing studies where pathogenicity was not assessed in the original publication, is in Supplementary Table S3, with reported phenotypes in Supplementary Table S4. We also note that one unpublished likely pathogenic variant in ClinVar, #1098346, has informative criteria provided (c.866C>T, p.(P289L), de novo occurrence without reported confirmation of maternity/paternity).

Four pathogenic/likely pathogenic missense variants had functional data supporting pathogenicity, but the effects on FMRP function differed between variants. Nonsense and frameshift variants were observed in patients with neurological and physical features of FXS and absent *FMR1* mRNA, presumably due to nonsense-mediated decay. Furthermore, one frameshift variant (c.1610dup) that did not completely eliminate *FMR1* mRNA produced a truncated protein that was still nonfunctional; it inappropriately localized to the nucleus due to a nuclear localization signal in the frameshifted C-terminus and produced novel axonal abnormalities in *Drosophila* [80].

### 3.4. Noncoding Small Variants

Noncoding variants from the promoter to 3′UTR were reported with varying levels of evidence for disease association (Table 4). Many of the variants initially reported in association with disease are relatively common in genomes from gnomAD, suggesting that they are benign. For brevity, Table 4 shows only noncoding variants initially reported as possibly disease-causing. Supplementary Table S5 contains all published variants and clinical evidence for impact with phenotypes in Supplementary Table S6.

There were no promoter or UTR variants with definite pathogenicity. Five pathogenic splicing variants were reported, including three at canonical splice sites, one at the end of exon 8, and one activating a cryptic splice site in intron 5. However, two variants initially thought to have pathogenic splicing effects, c.879A>C and c.990+14C>T, now have conflicting evidence (Table 4).

No variants have been reported to cause disease through effects on alternatively spliced transcript isoforms. One alternatively spliced transcript with the inclusion of a novel exon 9a, leading to a frameshift that escapes nonsense-mediated decay, was found in the course of evaluation of a patient with an FXS-like phenotype but is also present in multiple control individuals [91].

## 4. Discussion

### 4.1. Clinical Presentations of Pathogenic Non-Repeat Expansion Variants

**Figure 1:**
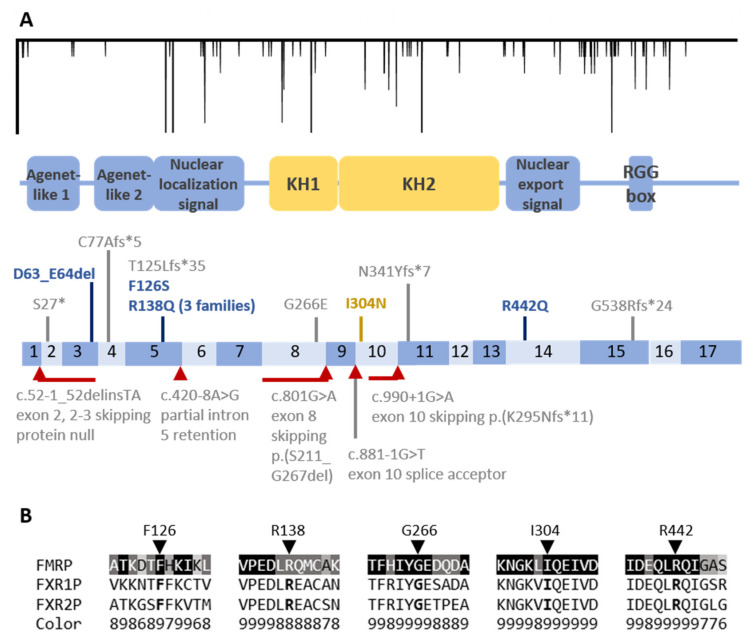
(**A**) Pathogenic and likely pathogenic small variants in *FMR1* in relation to exons and protein functional domains. The *FMR1* coding sequence (c.1-1899) is shown to scale with functional domain locations from the work of [95]. At the top, genetic variation in the general population is graphed with the x-axis showing c. locations and the y-axis showing the total minor allele frequency of missense and canonical splice variants in gnomAD (with higher frequencies extending farther downward, with several common variants clipped to a maximum frequency of 5 × 10 ^−5^.) Below, protein domains and exons 1–17 (dark and light boxes) are shown with the 11 coding variants above and 5 splice variants below the exon schematic. (**B**) Protein sequence conservation of the 5 residues involved in pathogenic missense variation. For each location, 11 aa of FMRP sequence around the residue is shown aligned to the paralogous FXR1P and FXR2P sequences. The FMRP sequence is color-colored based on evolutionary conservation from 1 (variable) to 9 using Consurf.

Deletion, missense, nonsense, frameshift, and splice variants were all identified as pathogenic in affected individuals. Figure 1 shows their locations in relation to functional domains of FMRP [92,93,94,95]. Consistent with the mechanism of pathogenesis, variants expected to completely eliminate FMRP production (whole-gene deletions, nonsense, frameshift, and frameshifting splice variants) resulted in similar phenotypes to full mutation alleles. They were associated with neurological and at least some physical features of FXS in males, while affected heterozygous females had primarily neurological involvement, which was generally milder than in their sons, and some were ascertained due to these sons rather than their own phenotypes. Patients with pathogenic missense variants, both those impairing FMRP’s canonical role in translational regulation and those affecting other protein functions and promoter-region deletions, were also reported to have some physical features of FXS (Supplementary Tables S1 and S2). However, this may be biased by several studies’ using the presence of FXS characteristics as a recruitment criterion, as well as the greater likelihood of testing *FMR1* in a patient with clinical features suggestive of FXS. The inclusion of *FMR1* sequence analysis in gene panels and exome slices (exome-based tests that only analyze a selected group of genes known to be associated with a particular phenotype) for less specific indications such as ID and ASD might broaden the range of missense and deletion phenotypes as more patients are reported.

While the loss-of-function variants were fully penetrant in males, the allele frequency of the R138Q missense variant suggests incomplete penetrance. Seven hemizygous males with this variant are present in gnomAD v.3.1.1, with an allele frequency among ethnic groups of up to ~1/1300 (Latino). On the other hand, the pathogenicity of the R138Q variant is well supported by functional studies, including a knock-in mouse model [70,72]. Three other pathogenic missense variants (G266E, I304N, R442Q) are absent in the general population. This raises the possibility that the R138Q phenotype is more amenable to modification by other genetic or environmental factors due to its different mechanism of pathogenicity.

The R138Q variant interferes with presynaptic functions of FMRP rather than its role in translational regulation at polyribosomes [70]. In contrast, the other three characterized variants interfere with FMRP’s role in translational regulation at polyribosomes: the G266E and I304N variants impair binding to polyribosomes and negative regulation of local protein synthesis (measurable as excessive AMPA receptor internalization in response to metabotropic glutamate receptor 5 signaling), while the R442Q variant protein inappropriately localizes to the nucleus [74,76,78,96]. These effects on function are consistent with the location of G266 and I304 within K-homology RNA-binding domains and of R442 in the nuclear export signal. In summary, all five of the pathogenic missense variants involve conserved residues within established functional domains. The identical amino acid is present in both human autosomal paralogs of FMRP (FXR1P and FXR2P) and is highly conserved among different species (Figure 1B).

Our analysis of deletion-related phenotypes (Table 1) was limited to deletions with breakpoints in or near *FMR1*. In clinical practice, microdeletions often extend into adjacent genes that also cause intellectual disability, especially *AFF2* located 550 kb downstream and *IDS* (mucopolysaccharidosis II) 1.5 Mb downstream. These larger deletions can present with additional clinical features related to the neighboring genes, as in proximal deletions including *SOX3* [97] and distal deletions including *IDS* [98,99,100,101,102,103,104,105,106,107].

Small duplications of the entire *FMR1* gene were also reported in possible association with disease in four patients where the duplicated regions only included *FMR1* and a gene of no known function, *FMR1NB*. Of these, two were males ascertained due to seizures, but clinical information was only available for one with myoclonic and absence epilepsy, who also had speech and motor delay, behavior problems, and fifth finger clinodactyly. Two heterozygous females had developmental delay and other syndromic features. Larger duplications have been reported in patients with different syndromic features that were hypothesized to be from other genes in the duplicated region [108,109].

### 4.2. Noncoding Variants Can Cause Disease, but New Variants Require Functional Confirmation

Pathogenic noncoding variants are a mechanism of *FMR1*-related disease, with five pathogenic splice variants described (Table 4). However, conflicting evidence for two variants reported as pathogenic indicates a need for caution and the use of multiple functional studies in interpreting new putative splice variants. The c.879A>C (IVS9-2, p.(V293=)) and c.990+14C>T variants were initially reported to be pathogenic due to detection of abnormally spliced transcripts, respectively by cDNA subcloning and by RT-PCR product sequencing [81,86]. Subsequently, another patient with the c.879A>C had no splicing or protein abnormalities, both variants were found in hemizygotes in gnomAD, and the c.990+14C>T variant turned out to be a common polymorphism in the general population, excluding its pathogenicity [27,89]. Protein analysis may thus be needed to determine whether any given splice variant truly alters FMRP in the patient being evaluated.

Interestingly, three promoter variants identified in a male DD sequencing cohort impaired transcription in a reporter gene expression assay yet are present in multiple hemizygotes in gnomAD [69]. Similarly, one 3′ UTR variant (c.*746T>C) found in affected half-brothers had clear negative effects on expression in multiple functional studies but is common in gnomAD with 72 hemizygotes [90]. This suggests that decreased transcription and translation of *FMR1* may not be fully penetrant or that the range of phenotypic expression includes mild effects on learning and behavior, perhaps analogous to the males with unmethylated full mutation alleles who have very mild phenotypes.

Several additional variants in the promoter region were identified due to alteration of restriction sites on Southern/RFLP testing or disruption of primer binding sites interfering with PCR assays but could be classified as benign based on functional data and/or population frequencies. Benign deletions and duplications in the 5′UTR/promoter region also occurred [34,35,40].

### 4.3. Rearrangement Breakpoints around the CGG Repeat

Expanded CGG repeats are known to be mutagenic, and deletion breakpoints in humans have previously been noted to cluster around the CGG repeat [8,9,18]. Consistent with this, many deletion events extending outside the repeat region were described, both de novo constitutional deletions and somatic mosaicism in patients with repeat expansion alleles. Besides their potential to affect *FMR1* expression and cause or modify FXS-related phenotypes, these deletions can impact routine laboratory testing for CGG repeat expansion in *FMR1*.

Like the small variants discussed above, deletions overlapping PCR amplicons or restriction sites are rare potential causes of allele misclassifications. Primer locations for PCR may vary between testing laboratories and are generally selected to avoid deletion hotspots as per the most recent ACMG technical standard [4]. For instance, the primer sets cited as examples in the above standard bind to the reference sequence at c.-165_-145/c.-15_+8 (with two mismatches in the forward primer used in one study) [110,111], c.-165_-145/c.-64_-44 [111], c.-250_-221/c.+2_29 [112], and c.-252_-235/c.-20_+6 [113,114]. Any of the whole-gene deletions or other large deletions overlapping at least one primer binding site (Table 1; Supplementary Tables S1 and S5) would produce a failure of amplification. On the other hand, smaller deletions overlapping these primer binding sites are also known to occur and are not necessarily pathogenic, for example, c.-4_+1delGAAGA (Supplementary Table S5), which was detected due to amplification failure with one set of primers and found in a male proband with normal FMRP levels [115]. Deletions wholly within the PCR amplicon could theoretically lead to inaccurate calculated CGG repeat sizes. For example, since full mutations can fail to amplify due to the length of the repeat, some individuals with mosaicism for full mutation and deletion show only the small PCR product originating from the deleted allele [45].

Deletions including part of an expanded CGG repeat can reduce the size of the repeat, restoring expression of the gene, and normal *FMR1* expression can be observed even if some of the adjacent 5′ UTR sequences outside the repeat is deleted [35]. However, the fact that several of the de novo constitutional deletions occurred on alleles without repeat expansions suggests that the CGG repeat may be unstable even at normal sizes. Such deletions, if limited to the 5′ UTR or wholly within the CGG repeat, can reduce the number of AGG interspersions in the repeat without disrupting *FMR1* function, producing alleles that are not associated with disease but have a higher probability of expansion [7]. Conversely, since AGG interspersion reduces the chance of repeat expansion, it would be interesting to test whether it also inhibits de novo deletion events, i.e., whether deletions are associated with fewer AGGs in maternal alleles. This information was not available in many of the reported cases but could be collected in the future as more laboratories perform CGG repeat tests that include the determination of AGGs.

Mosaicism is increasingly appreciated as a factor in the occurrence and variability of genetic disease, with mosaic pathogenic variants found in up to 8% of affected probands with other X-linked neurodevelopmental disorders such as *CDKL5* and *PCDH19* epilepsy [116]. Besides mosaic *FMR1* variants in individuals without repeat expansions, mosaic deletions occur in patients with full mutations but are of uncertain clinical importance due to the small number of characterized patients and presence of additional mosaicism for premutations. A few patients had discordant phenotypes from non-mosaic siblings. One had the Prader-Willi syndrome-like presentation, while his non-mosaic brother had classic FXS [29,46,57]. Two other patients with mosaicism for deletion, premutation, and full mutation alleles had milder FXS and higher cognitive functioning than expected [56,59]. These individuals had 18–22% of wild-type FMRP expression in blood. Another patient with mosaicism for a deletion and full mutation allele, without a reported premutation allele, had typical FXS despite FMRP staining in 28% of lymphocytes [61]. Therefore, although constitutional deletions of the CGG repeat can be functional alleles as discussed above, it is currently difficult to separate any contribution of deletion alleles from that of unmethylated premutation alleles in mosaic full mutation patients. Furthermore, many such mosaic deletions may be undetected due to technical factors such as occurrence outside PCR amplicons used for testing. Using a relatively large 557 bp amplicon, Gonçalves et al. reported relatively frequent detection of mosaic deletions in the presence of full mutations (2.02% of a cohort referred for FXS testing, vs. 8.09% found to have full mutation only) [45].

Two large studies indicate the approximate frequency of deletion alleles. A multicenter study on 1105 families (5062 unique individuals tested) with members diagnosed with FXS by laboratories in Spain identified three deletion alleles [117]. In the general population, two deletion alleles were identified in a carrier screening study of 20,188 asymptomatic pregnant women in Taiwan [33]. One woman was heterozygous for a deletion of the 5′UTR; the other was heterozygous for a full mutation allele, but testing of her male fetus showed an exon 1 deletion. The screening test used was CGG repeat-primed PCR, which would only be expected to observe deletions close enough to exon 1 to be within the PCR product. Therefore, the frequency of deletion alleles in the general population might be higher.

### 4.4. Detection of FMR1 Variants Other than Repeat Expansions

While most patients with FXS are expected to have full mutation size CGG repeat expansion in the 5′ UTR of *FMR1*, a negative CGG repeat expansion on targeted testing does not completely exclude the diagnosis of FXS [65,71]. In individuals with features suggestive of FXS and no pathogenic CGG repeat expansion identified, testing methods investigating *FMR1* sequence variants are recommended. The potential for genomic sequencing to simultaneously interrogate *FMR1* together with several other genes in the same assay makes this strategy an ideal follow-up testing approach in these patients [77]. Clinical genomic sequencing can either be targeted with the use of multigene panel testing or more comprehensive with exome sequencing (ES) or genome sequencing (GS). Custom multigene panels, including *FMR1* and other genes of interest with integrated deletion/duplication analysis, can allow for effective identification of coding and targeted noncoding single nucleotide variants (SNVs) and intragenic CNVs. ES or GS, on the other hand, can help identify coding and potentially deep intronic (applicable to GS) *FMR1* sequence variants [77]. While applications of long-read next-generation sequencing (NGS) technologies have shown great promise in reliably detecting structural variations, non-diagnostic short read-based ES or GS may require follow-up testing methods such as exome array to screen for large exon-level or whole-gene CNVs not reliably detected by current clinical genomic sequencing methods [118,119].

Finally, the architecture of *FMR1* commends special considerations when analyzing and interpreting the results of genomic sequencing. First, both normal-sized and expanded CGG repeat alleles tend to be unstable, with many deletion breakpoints clustering around the repeat region. Second, short read NGS technologies and single-end sequencing methods face the issues of poor sequencing or sequencing bias across GC-rich genomic regions and inadequate mapping of tandem repeat sequences [118], all the things that currently make it challenging not only to detect and size CGG repeat expansion but also to identify potential sequence variants within or around the repeat region. These factors can be mitigated for NGS tests through higher coverage and the use of bioinformatic tools capable of analyzing in-repeat reads, allowing information to be obtained about expansions longer than the read length [120,121].

## Figures and Tables

**Table 1:** *FMR1* CNVs reported in the absence of CGG repeat expansions ^a^.

Ref.	Location ^b^			Number Affected with Variant, Sex (M/F)	Inheritance/Originating Allele	Ascertainment	ACMG Criteria [12,13]	Conclusion
	Proximal	Distal	Type					
[15]	>140198205 (DXS1232-DXS105), <7 Mb upstream	<148215436 (between 141R-DXS533), 264 kb downstream	Del	1M	Maternal (unaffected), de novo in mother (both grandparents lack deletion with normal CGG repeat counts)	ID, obesity	CNV 2A 5G_0.1	PATH (1.1)
[16]	>140784366 (CDR1-sWXD2905), <7 Mb upstream	<148203554 (DXS7847); >DXS8318, <252 kb downstream	Del	1M + mother	Maternal (mildly affected)	DD, clinical suspicion	CNV 2A 5G_0.1	PATH (1.1)
[17] #3	142254456, 5.7 Mb upstream	148191426, 240 kb downstream	Del het	1F	De novo	Epilepsy research database	CNV 2A 5A_0.15	PATH (1.15)
[18]	147158490, 753 kb upstream	148171882, 221 kb downstream	Mosaic del (90%)	1M	Mosaic in proband on 23-repeat allele (mother 23, 30 repeats) with breakpoints in LINE1 elements	ID	CNV 2A 5A_0.15	PATH (1.15)
[19]	147630212–147721930 (DXS532–DXS548), 200–300 kb upstream	Intragenic, ~30 kb downstream of HTF island	Del	1M	De novo	ID, facies, testicle size	SVI PVS1_Strong PS2 PM2	PATH
[20]	147653688 ^c^, 260 kb upstream	147955394, 4 kb downstream	Del	1M	Mosaic mother (3/400 lymphocytes) with same deletion in subsequent pregnancy	Clinical FXS	CNV 2A 5H_0.1	PATH (1.1)
[21]	>147722126 (DXS548); >DXS477, <cosmid 494; <190 kb upstream	147932685–147932763, exon 9	Del	1M	? (mother “borderline intelligence”)	ID	SVI PVS1_Strong PM2	LPATH
[22] #2	>147722126 (DXS548); >G9L, <FRAXAC1; <190 kb upstream	147936614–147937465, intron 10	Del	1M	De novo on 19-repeat allele (mother 19, 51 repeats)	ID, clinical FXS	SVI PVS1_Strong PS2 PS3 PM2	PATH
[23]	<147722126 (includes DXS548), not mapped further; >190 kb upstream	?, includes *FMR1*	Del	1M + infant brother	Mat germline mosaicism (2 brothers, absent in mother) (mother has congenital digit amputations)	DD	CNV 2A 5G_0.1	PATH (1.1)
[24] #18072	147787231, 125 kb upstream	148041310, 90 kb downstream	Del	2M	Maternal (unaffected het)	Autism cohort	CNV 2A5G_0.1	PATH (1.1)
[25] #3	147838064, 74 kb upstream	148103912, 400 kb downstream	Del	1M	De novo	Clinical lab sample	CNV 2A 5A_0.15	PATH (1.15)
[26]	15–80 kb upstream (G9L YAC)	147930245–147932424, intron 7	Del	1M	De novo (normal repeat alleles in mother)	DD	SVI PVS1_Strong PS2 PS3 PM2	PATH
[27,28]	4.4 kb upstream	194 kb downstream	Del	1M	Maternal (unaffected mosaic) ^d^; breakpoints in L1MC2 and MIR3 elements	Preschool cohort with ID and >=1 FXS feature	CNV 2A 5G_0.1	PATH (1.1)
[29] #24	147910365, 1.5 kb upstream	147912050 (within CGG repeat, no AGG interspersions in remaining sequence)	Del	4M, 2F	Maternal (unaffected)	Speech delay, hyperactivity	CNV 2C2_0.3 5D_0.3	VUS (0.6)
[22] #1	147911457, 462 bp upstream of transcription start	147912135, c.-45 (including entire CGG repeat)	Mosaic del (40%)	1M	De novo	Epilepsy, other clinical	CNV 2C2_0.3 5A_0.15	VUS (0.45)
[30]	~147911751 (~300 bp upstream of CGG)	? (~400 bp size deletion)	Del	1M	De novo (mother is het for 700–900 repeat full mutation)	ID, aggressive behavior	CNV 2C2_0 5A_0.15	VUS (0.15)
	Also has 13p+ polymorphism							
[31]	147911831, 88 bp upstream of transcription start	147912185, c.6	Del	1M		ID male with >=2 FXS features	SVI PVS1_Moderate PS3 PM2	LPATH
[32]	147911966, c.-214	Within CGG repeats, 19 remaining (no AGGs)	Del	N/A (M)	Maternal mosaic (unaffected het for 430–530 repeat full mutation, deletion occurred on full mutation allele by haplotype)	Unaffected, prenatal testing	CNV 2C2_0 5F_0	VUS (0)
[33]	147911981, c.-199	147912050 (within CGG repeats, 9 remaining)	Del het	N/A	Transmitted from unaffected female proband to male fetus	Unaffected individual, population screening study	CNV 2C2_0 5F_0	VUS (0)
[34]	147911984, c.-196	147912140, c.-40 (distal to CGG repeat)	Del het	N/A (F)	De novo, from maternal full mutation allele	Asymptomatic; due to brother with FXS	CNV 2C2_0 5A_-0.3; lymphoblasts are FMRP+	VUS (−0.3) with additional benign evidence
	*In trans* with large deletion involving FMR1-46,X,del(X)(q24)							
[35]	147912024, c.-156	147912111, c.-69 (1 bp distal to CGG repeat)	Del	N/A (M)	De novo, from maternal full mutation allele	Asymptomatic; due to maternal full mutation	CNV 2C2_0 5A_-0.3 (unaffected); normal blood *FMR1* mRNA level	VUS (−0.3) with additional benign evidence
[36] APN26, [37] #1	<147948682 (Deletion of exon 17)	>147964837 (Deletion of exon 17)	Del	3M	Maternal (unaffected)	ID sequencing cohort	SVI PVS1_Moderate PS3_Supporting PM2 PP1	LPATH
[33]	Undetermined 5′ UTR	Loss of exon 1	Del	1M fetus	Mother is full mutation (280 repeat) het	Population screening study	SVI PVS1_Strong PS3	PATH
[38] #3660	Intragenic (~197 bp flanking CGG repeat in exon 1)	Intragenic	Del	1M	? (mother has mild ID)	ID cohort	CNV 2C2_0 5G_0.1	VUS (0.1)
**Potentially including *AFF2* (additional reports of confirmed *FMR1-AFF2* deletions not shown)**								
[25] #4	142773651, 5.1 Mb upstream	148673162, 722 kb downstream	Del het	1F	De novo	Clinical lab sample	CNV 2A 5A_0.15	PATH (1.15)
[17] #2	143096757, 4.8 Mb upstream	149186971, 1.2 Mb downstream	Del het	1F	De novo	Epilepsy research database	CNV 2A 5A_0.15	PATH (1.15)
**Duplications**								
[25] #1	147707513 (204 kb upstream)	148070742 (120 kb downstream)	Dup het	1	De novo	Clinical testing (lab)	CNV 2H_0 4C_0.15x3 5A_0.15	VUS (0.6)
[25]	147709189 (203 kb upstream)	147976294 (25 kb downstream)	Dup	1		Clinical testing for seizures (lab)	CNV 2H_0 4C_0.15x3 5A_0.15	VUS (0.6)
[25] #2	147796927 (115 kb upstream)	148144967 (194 kb downstream)	Dup het	1	De novo	Clinical testing (lab)	CNV 2H_0 4C_0.15x3 5A_0.15	VUS (0.6)
[39]	147894723 (17 kb upstream)	147979356 (28 kb downstream)	Dup	1	De novo (Proband also has small 1q44 paternal dup and 4p15 del)	Myoclonic seizures	CNV 2H_0 4C_0.15x3 5A_0.15; normal mRNA in leukocytes	VUS (0.6)
[40]	147912003 (c.-177)	147912051 (c.-129)	Dup	N/A (0.2–0.3% X chromosomes in study)		Finnish population samples with multiple unaffected males	CNV 2I_0 4O_-1	BEN (−1)

^a^ In addition to the listed CNVs, we note that (1) two deletion alleles extending into the CGG repeat region from g.147911980 and g.147912003 were identified in a sequencing cohort without definite information regarding whether they were mosaic, constitutional, or associated with a CGG repeat expansion [41]; and (2) we were unable to access the full text of one report of a deletion in an affected patient in the Ukrainian-language literature (PubMed 9381553). ^b^ Breakpoints listed as approximate (~) were specified in relation to other sequence landmarks by the authors. ^c^ Corresponding to the hg19 coordinates 146,735,206-147,036,914 given for the aCGH result; the GRCh38 coordinates and ClinVar entry given in the same publication appear to be for a different region on chr6. ^d^ Mosaic for deletion in 4% alleles in blood, 8% skin, 11% urine sediment, 12% menses, 33% eyebrow by quantitative polymerase chain reaction (PCR); 4/1000 leukocytes, 213/1600 fibroblasts by FISH. #: Case number of proband in report, if given; ~: approximately; PATH: pathogenic; LPATH: likely pathogenic; ID: intellectual disability; DD: developmental delay; Del: deletion; Dup: duplication; het: heterozygous; N/A: not applicable; M: male; F: female; FXS: fragile X syndrome; ?: unclear.

**Table 2:** Mosaic deletions extending outside the CGG repeat region in patients with *FMR1* repeat expansions.

Ref./ Case #	Breakpoints ^a^		c.		Sex	Expansion	% Deletion in Blood	Functional/ Phenotypes ^b^	Maternal CGG Repeats
	Proximal	Distal	Proximal	Distal					
[48]	147911612	147912529	upstream	c.51+299	M	300–350		No FMRP (blood, hair roots)	~100, normal
[45] #1513	~147911695	~147912736	upstream	~c.51+506	M	FM		3–4% mRNA	99/105, 30
[49]	147911883	147912368	upstream	c.51+138	M	FM	20–30%		
[50] #12	147911908(ins(9))	147912135	upstream	c.-45	M	250, 510, 710; 170–320 with deletion	9% (with 22.5% unmethylated, 68.5% methylated)	11% FMRP, 72% mRNA; Mullen early learning composite 90	
[51] #1	~147911938	CGG repeat	~c.-242	30 repeats	M	300–500	17%		156, 23
[50] #11	147911941	147912291	c.-239	c.51+61	M	420, 470, 1040; 84 with deletion	20% (with 13% unmethylated, 67% methylated)	6% FMRP, 17% mRNA; IQ 66	
[52] #6	~147911945	147912267	~c.-235	c.51+37	M	FM		No FMRP (lymphocytes)	FM, normal
[50] #9	147911960 (insTT)	147912133	c.-220	c.-47	M	350, 510, 630, 1050; 150–500 with deletion	13% (with 28% unmethylated, 59% methylated)	32% FMRP, 64% mRNA; IQ 47, autism spectrum disorder	
[53]	~147911964	~147912164	~c.-216	~c.-16	M	70, 463–846		FXS + FXTAS 97% FMRP (blood)	PM/FM mosaic, normal
[52] #5	~147911966	147912125 ins(7)	~c.-214	c.-55	M	FM		No FMRP (lymphocytes)	FM, normal
[46] #2	147911966	147912135	c.-214	c.-45	M	FM	5–10%		PM, normal
[47]	147911966	147920712	c.-214	c.51+8482	M	~300	90%; 85% fibroblasts (12/14)		FM, normal (affected)
[54]	147911976	147912202	c.-204	c.23	M	58–60, FM	2% (23% PM, 75% FM)		
[46] #4	147911978	147912134	c.-202	c.-46	M	FM	5–10%		
[46] #3	147911978	~147912545	c.-202	~c.51+315	M	FM	15%		
[55]	147911988	147912198	c.-192	c.19	F	~400/normal het	21% (29% FM, 27% methylated normal, 23% unmethylated normal)		PM, normal
[50] #10	147911990 (insCG)	147912138	c.-190	c.-42	M	450, 540, 620, 930, 1080; 118 with deletion	4% (with 14% unmethylated, 82% methylated)	5% FMRP, 14% mRNA; IQ 69	
[56]	~147911990	~147912137	~c.-190	~ c.-97	M	170, 230, 450	15% (60% methylated FM, 25% unmethylated PM)	18% FMRP (lymphocytes), 6% FMRP (hair roots), mild (IQ 81)	PM, normal
[46] #1, [57] #2, [58]	147911998	147912288	c.-182	c.51+58	M	FM	5–10%	Prader-Willi syndrome phenotype	PM, normal
[59]	147912010	147912111	c.-170	c.-69	M	165, >200	53% (Southern; with 37% PM, 10% FM)	22% WBCs are FMRP+ Milder FXS than non-mosaic brother	109, normal
[60]	147912020	147912171	c.-160	c.-9	M	FM			
[45] #1629	147912023	147912115	c.-157	c.-65	M	FM			PM, normal
[45] #1033	147912023	147912150	c.-157	c.-30	M	FM		3% mRNA	99/106, 31
[45] #1337	147912039	147912110 insCTGGG	c.-141	c.-70	M	FM		16–19% mRNA	79, 30
[45] #1234	147912044	147912136	c.-136	c.-44	M	FM		4–6% mRNA	174/>200, 36
[61]	~147912060	147912140	~c.-120 (4th CGG)	c.-40	M	FM	20–30%	28% lymphocytes are FMRP+ Typical FXS	
[52] #7	>147909424 (RFLP)	<147912612 (RFLP)	upstream	<c.51+382	M	FM		No FMRP (lymphoblastoid cell line)	PM, normal
[62]	>147911959 (primer)	<147912181 (primer)	>c.-221	<c.2	M	FM			
[52] #8	? (RFLP)	? (RFLP)	entire repeat		M	FM			FM, normal

^a^ Breakpoints listed as approximate (~) were specified in relation to other sequence landmarks by the authors. ^b^ As % of wildtype levels, by quantitative reverse transcriptase PCR (qRT-PCR) for mRNA. #: case number of proband in report, if given; c.: coding DNA position; FM: full mutation; PM: premutation; specific repeat lengths shown if given in original publication.

**Table 3:** Missense, nonsense, and frameshift *FMR1* variants reported as pathogenic or possibly disease-causing.

Ref.	Location		Number Affected with Variant, Sex (M/F)	Other Variants in Patient	Inheritance in Proband	ACMG Criteria [11]	Conclusion
	c.	p.					
[64]	c.80C>A	p.S27*	1M, 1F		Mat (affected)	PVS1 PS3 PM2 PP1	PATH
[65]	c.188_193del	p.(D63_E64del)	1M		De novo confirmed	PS2 PM2 PM4 PP3	LPATH
[66]	c.229delT	p.(C77Afs*5)	1M	Maternal PATH 308 kb del involving *PTCHD1-AS* (autism)	De novo confirmed	PVS1 PS2 PM2 BP5	PATH
[67] #1	c.373delA ^a^	p.(T125Lfs*35)	1M		De novo	PVS1 PS3 PM6	PATH
[37,68] ^b^	c.377T>C	p.(F126S)	1M	*KDM5C* I1349M (maternal), *ALG13* H771R (maternal)	De novo	PS2 PM6 PP3	LPATH
[69,70]	c.413G>A	p.R138Q	1M, 1F		Maternal (affected, learning disability/anxiety)	PS3 PP1 PP4; note 7 hemizygotes in gnomAD v3 exomes (rs200163413)	LPATH
[71]	c.413G>A	p.R138Q	1M	(1st cousin consanguinity)	Maternal		
[72]	c.413G>A	p.R138Q	N/A				
[73]	c.413G>A	p.R138Q	1F		Absent in mother; healthy father not tested		
[74]	c.797G>A	p.(G266E)	1M		Maternal (unaffected)	PS3 PM2 PP3	LPATH
[75,76]	c.911T>A	p.(I304N)	1M	Known PHKA2 deficiency (glycogen storage disease IXa1)	De novo confirmed	PS2 PS3 PM2 PP3	PATH
[77] #1	c.1021–1028delinsTATTGG	p.N341Yfs*7	2M		Mother not tested; had epilepsy	PVS1 PS3 PP1	PATH
[78]	c.1325G>A	p.(R442Q)	2M + mother, grandmother	11 kb paternal deletion of part of *MYH7*	Maternal (affected)	PS3 PM2 PP1 PP3	LPATH
[79]	c.1444G>A	p.(G482S)	1M			PM2 BP4	VUS
[63]	c.1550C>T ^c^	p.(P517L)	1M		?	PM2	VUS
[79]	c.1601G>A	p.(R534H)	2M (unrelated), 1F		Maternal (unaffected) in both families	PM2	VUS
[80]	c.1610dup	p.(G538Rfs*24)	1M		?	PVS1 PS3 PM2	PATH
[81] TN351	c.1637G>A	p.(R546H)	1M	*FMR1* c.990+14C>T VUS	?	PM2	VUS

^a^ New BglII restriction site. ^b^ All references in literature are to a variant identified in the DDD study that was not listed in that publication but corresponds to DECIPHER patient #259197. ^c^ Reported as NM_001185081.1:c.1216C>T (p.(Q406*)) on an alternate reading frame from the reference transcript. #: case number of proband in report, if given; M: male; F: female; N/A: not applicable; ?: unclear; LPATH: likely pathogenic; PATH: pathogenic.

**Table 4:** Noncoding *FMR1* variants reported as pathogenic or possibly disease-causing.

Ref.	Location		Number Affected with Variant, Sex (M/F)	Inheritance in Proband	Patient and/or Functional Data	ACMG Criteria	Conclusion
	DNA	r./p.					
[69]	“c.-332G>C” (promoter)		1M (male DD cohort)		Reduced reporter expression to 5.9% of WT (10 hemizygotes in gnomAD, rs922007219)	BS1 BS2 PS3	VUS
[69]	“c.-293T>C” (promoter)		1M (male DD cohort)		Reduced reporter expression to 29.2% of WT(7 hemizygotes in gnomAD, rs1222840333)	BS1 BS2 PS3	VUS
[69]	c.-254A>G		1M (male DD cohort)		Reduced reporter expression to 36.2% of WT(8 hemizygotes in gnomAD, rs1217601043)	BS1 BS2 PS3	VUS
[82]	CGG repeat 26 of 31 CGG>CCG	N/A (new EagI site)	1M	Maternal (unaffected)	Normal % of FMRP+ lymphocytes, but statistically significant decrease to 76% normal FMRP level in lymphoblastoid cell line	None	VUS
[83]	c.18G>T	p.(V6=)	2M (unrelated)	Maternal (unaffected) in 1	Normal intron 1 splicing in lymphoblastoid cell line (1 patient), normal FMRP Western blot in lymphoblastoid cell line (1 patient)	BS1 BS2 BS3	BEN
[79]	c.18G>T	p.(V6=)	4/508 male ID/DD cohort				
[69]	c.18G>T	p.(V6=)	13 samples, 19 controls		Observed in multiple unaffected controls		
[67]	c.52-1_52delinsTA	2 abnormal transcripts	1M + mother	Maternal (affected)	No normal transcripts; 2 abnormal transcripts with skipped exon 2 and skipped exons 2–3; FMRP absent in lymphoblastoid cell line	PVS1 PS3 PP1	PATH
[37] #3	c.420-8A>G	r.419_420ins420-7_420-1 (p.(M140Ifs*3))	1M (ID cohort)	Maternal (unaffected, no XCI skewing)	Cryptic splice acceptor leading to retention of 7 nt from intron 5 (blood)	PS3 PM2	LPATH
[84,85]	c.801G>A (IVS8-1)	r.631_801del (p.(S211_G267del)); exon 8 skipping	1M (ID cohort)	De novo with maternity/paternity confirmed	No family history of DD (parental first cousin consanguinity) Exon 8 skipping with no normal RT-PCR product; rat model with deletion of exon 8 is affected	PS2 PS3 PM2	PATH
[86]	c.879A>C (IVS9-2)	p.(V293=); reported abnormal splicing intron 9	1F (autism/ID cohort)		Inclusion of intron 9 sequence in 23/36 subclones from peripheral blood cDNA	BS2	VUS
[27]	c.879A>C (IVS9-2)	p.(V293=); no splicing abnormality found	1M (ID, 1 FXS feature)	Maternal (unaffected)	No abnormal splice amplicons found; normal exon 9–10 junction in RT-PCR product sequence in blood; FMRP present in blood homogenate (1 hemizygote in gnomAD, rs782013865)		
[77]	c.881-1G>T	? (exon 10 splice acceptor)	1M (clinical suspicion)	Maternal (1:99 skewed XCI, unaffected)		PVS1 PM2	LPATH
[37]	c.990+1G>A	r.881_990del (p.(K295Nfs*11)) (exon 10 skipping)	1M (ID cohort)	De novo	Exon 10 skipping in blood	PVS1 PS3 PM2 PM6	PATH
[69]	c.990+4T>C	? (intron 10)	1M (DD cohort)			PM2	VUS
[81]	c.990+14C>T	r.881_990del (p.(K295Nfs*11)) (exon 10 skipping)	3 unrelated males (ID, FXS feature cohort)		Exon 10 skipping on peripheral blood RT-PCR product sequencing in 2 probands (TN-183, TN-351)	BA1 BS2	BEN
[87]	c.990+14C>T		81 control individuals		Observed in many controls from general population		
[79]	c.990+14C>T		45/508 in ID/DD cohort				
[88]	c.990+14C>T		7M/4F among 88 patients with ASD		Statistically significant (*p* = 0.0123) higher frequency in ASD patients vs. controls Stably inherited in unaffected family members		
[89]	c.990+14C>T				No significant transmission disequilibrium (*p*=0.26) Allele frequency 65% (22/34) in East Asian controls; concluded that previously observed association with ASD was false positive due to population stratification (gnomAD AF > 10%, rs25714)		
[27]	c.*312_313dupT		1 (ID, 1 FXS feature cohort)			PM2	VUS
[69,90]	c.*746T>C		2M (proband and half-brother from DD cohort)		Faster mRNA decay with FMRP level 80% of normal in lymphoblastoid cell line; variant sufficient and necessary to decrease reporter gene expression in vitro; loss of metabotropic glutamate receptor-stimulated upregulation of reporter expression in transfected mouse neurons (72 hemizygotes in gnomAD, rs183130936)	PS3 PP1 BS1 BS2	VUS

c.: coding DNA position; r.: RNA position; p.: protein position; ASD: autism spectrum disorder; ID: intellectual disability; DD: developmental delay; N/A: not applicable; M: male; F: female; AF: allele frequency; WT: wildtype; BEN: benign.

## Data Availability

The data presented in this study are available in Supplementary Tables S1–S6 of this article.

## Supplementary Materials

The following are available online at https://www.mdpi.com/article/10.3390/genes12111669/s1, Table S1: Information supporting pathogenicity criteria for CNVs in Table 1; Table S2: Phenotypes of individuals reported with *FMR1* CNVs; Table S3: All published coding region small variants; Table S4: Phenotypes of individuals reported with *FMR1* coding variants; Table S5: All published noncoding small variants; Table S6: Phenotypes of individuals reported with *FMR1* splice/noncoding variants.
