# Supplementary material for: Beyond Trinucleotide Repeat Expansion in Fragile X Syndrome: Rare Coding and Noncoding Variants in FMR1 and Associated Phenotypes
Source: Genes (Basel). 2021 Oct 22;12(11):1669. doi: 10.3390/genes12111669 (PMC8623550; doi:10.3390/genes12111669)
Supplement: Supplementary file 1 [file genes-12-01669-s001.zip › Supplementary Table S1.pdf]

Supplementary Table S1. Additional details of CGG repeat testing in probands and information used to apply pathogenicity criteria for variants in Table 1.

Ref.	Location			# affected with variant, sex	CGG repeats	Other variants in proband	Inheritance/originating allele	Segregation	*FMR1* expression in patient	Ascertainment	ACMG criteria	Conclusion
	Proximal	Distal	Type									
[1]	>140198205 (DXS1232-DXS105), <7 Mb upstream	<148215436 (between 141R-DXS533), 264 kb downstream	Del	1M	Absent		Maternal (unaffected), origin de novo in mother (both grandparents lack deletion with normal CGG repeat counts)			ID, obesity	CNV 2A 5G_0.1	PATH (1.1)
[2]	>140784366 (CDR1-sWXD2905), <7 Mb upstream	<148203554 (DXS7847); >DXS8318, <252 kb downstream	Del	1M + mother	Absent		Maternal (mildly affected)			DD, clinical suspicion	CNV 2A 5G_0.1	PATH (1.1)
[3] #3	142254456, 5.7 Mb upstream	148191426, 240 kb downstream	Del het	1F	?		De novo			Epilepsy research database	CNV 2A 5A_0.15	PATH (1.15)
[4]	147158490, 753 kb upstream	148171882, 221 kb downstream	Mosaic del (90%)	1M	23 (weak signal)		Occurred on 23-repeat allele (mother has 23, 30-repeat alleles); breakpoints are LINE1 elements			ID	CNV 2A 5A_0.15	PATH (1.15)
[5]	147630212-147721930 (DXS532-DXS548), 200-300 kb upstream	Intragenic, ~30kb downstream of HTF island	Del	1M	no fragile site		De novo			ID, facies, testicle size	SVI PVS1_Strong PS2 PM2	PATH
[6]	147653688, 260 kb upstream	147955394, 4 kb downstream	Del	1M			Mosaic in mother (3/400 lymphocytes) & same deletion in subsequent pregnancy			Clinical FXS	CNV 2A 5G_0.1	PATH (1.1)
[7]	>147722126 (DXS548); >DXS477, <cosmid 494; <190 kb upstream	147932685-147932763, exon 9	Del	1M	Absent		? (mother "borderline intelligence")			ID	SVI PVS1_Strong PM2	LPATH
[8] #2	>147722126 (DXS548); >G9L, <FRAXAC1; <190 kb upstream	147936614-147937465, intron 10	Del	1M	no fragile site		De novo on 19-repeat allele (mother 19, 51 repeats)		mRNA absent (lymphoblastoid cell line RT-PCR)	ID, clinical FXS	SVI PVS1_Strong PS2 PS3 PM2	PATH

[9]	<147722126 (includes DXS548), not mapped further; >190 kb upstream	?, includes *FMR1*	Del	1M + infant brother	Absent	Mat germline mosaicism (2 brothers, absent in mother) (mother has congenital digit amputations)			DD	CNV 2A 5G_0.1	PATH (1.1)
[10] #18072	147787231, 125 kb upstream	148041310, 90 kb downstream	Del	2M		Maternal (unaffected het)	Present in both affected brothers in this family		Autism cohort	CNV 2A 5G_0.1	PATH (1.1)
[11] #3	147838064, 74 kb upstream	148103912, 400 kb downstream	Del	1M	no PCR product	De novo			Clinical lab sample	CNV 2A 5A_0.15	PATH (1.15)
[12]	15-80 kb upstream (G9L YAC)	147930245-147932424, intron 7	Del	1M	Absent	De novo (both maternal alleles normal repeat length)		FMRP absent (lymphoblastoid cell line)	DD	SVI PVS1_Strong PS2 PS3 PM2	PATH
[13], [14]	4.4 kb upstream	194 kb downstream	Del	1M	Absent	Maternal (unaffected mosaic); breakpoints are within L1MC2 and MIR3 elements			Preschool cohort with ID and >=1 FXS feature	CNV 2A 5G_0.1	PATH (1.1)
[15] #24	147910365, 1.5 kb upstream	147912050 (within CGG repeat, no AGG interspersions in remaining sequence)	Del	4M, 2F	~45	Maternal (unaffected)	Males: 4 affected/4 hemizygotes (#14, 15, 23, 24) and 0 affected/4 noncarriers (#5, 13, 18, 19); Females: 2 affected/7 het and 0 affected/3 noncarriers		Speech delay, hyperactivity	CNV 2C2_0.3 5D_0.3	VUS (0.6)
[8] #1	147911457, 462 bp upstream of transcription start	147912135, c.-45 (including entire CGG repeat)	Mosaic del (40%)	1M	no fragile site	De novo			Epilepsy, other clinical	CNV 2C2_0.3 5A_0.15	VUS (0.45)

[16]	~147911751 (~300 bp upstream of CGG)	? (~400 bp size deletion)	Del	1M	Absent	13p+ polymorphism (karyotype), father “mentally slow”	De novo (mother is FXS full mutation 700-900 repeat het, unaffected with 95% skewed XCI toward nl allele)		ID, aggressive behavior	CNV 2C2_0 5A_0.15	VUS (0.15)
[17]	147911831, 88 bp upstream of transcription start	147912185, c.6	Del	1M				FMRP absent (lymphoblastoid cell line)	ID male with >=2 FXS features	SVI PVS1_Moderate PS3 PM2	LPATH
[18]	147911966, c.-214	Within CGG repeats, 19 remaining (no AGGs)	Del	N/A (M)			Maternal (unaffected mosaic, het for 430-530 CGG full mutation); del on full mutation allele by haplotype		Unaffected, prenatal testing	CNV 2C2_0 5A_0	VUS (0)
[19]	147911981, c.-199	147912050 (within CGG repeats, 9 remaining)	Del het	N/A			Transmitted from unaffected female proband to male fetus		Unaffected individual, population screening study	CNV 2C2_0 5F_0	VUS (0)
[20]	147911984, c.-196	147912140, c.-40 (distal to CGG repeat)	Del het	N/A (F)	Absent	Large deletion involving FMR1 *in trans* (46,X,del[X][q24])	De novo, from maternal full mutation allele	FMRP present (lymphoblastoid)	Asymptomatic; due to brother with FXS	CNV 2C2_0 5A_-0.3	VUS (-0.3) with additional benign evidence
[21]	147912024, c.-156	147912111, c.-69 (1 bp distal to CGG repeat)	Del	N/A (M)			De novo, from maternal full mutation allele	mRNA level normal (whole blood qRT-PCR)	Asymptomatic; due to maternal full mutation	CNV 2C2_0 5A_-0.3 (unaffected)	VUS (-0.3) with additional benign evidence

[22] APN26, [23] #1	<147948682 (Deletion of exon 17)	>147964837 (Deletion of exon 17)	Del	3M		Maternal (unaffected)	Present in 3 affected brothers	Normal mRNA quantity but retained intron 16 sequence (1 brother) and 44 aa truncation of FMRP with 50% reduced level (2 brothers) (blood, lymphoblastoid cell line)	ID sequencing cohort	SVI PVS1_Moderate PS3_Moderate PM2 PP1	LPATH
[19]	Undetermined 5′ UTR	Loss of exon 1	Del	1 male fetus		Mother is full mutation (280 repeat) het			Population screening study	SVI PVS1_Strong PS3	PATH
[24] #3660	Intragenic (~197 bp flanking CGG repeat in exon 1)	Intragenic	Del	1M			?, FHx mild ID mother, psychiatric problems mother, 2 mat uncles with ID (no genotypes)		ID cohort	CNV 2C2(0) 5G_0.1	VUS (0.1)
**Potentially including *AFF2* (additional reports of confirmed *FMR1*-*AFF2* deletions not shown)**											
[11] #4	142773651, 5.1 Mb upstream	148673162, 722 kb downstream	Del het	1F		De novo			Clinical lab sample	CNV 2A 5A_0.15	PATH (1.15)
[3] #2	143096757, 4.8 Mb upstream	149186971, 1.2 Mb downstream	Del het	1F	?	De novo			Epilepsy research database	CNV 2A 5A_0.15	PATH (1.15)
**Duplications**											
[11] #1	147707513 (204 kb upstream)	148070742 (120 kb downstream)	Dup het	1		De novo			clinical testing (lab)	CNV 2H_0	VUS (0.6)

													4C_0.15x3 5A_0.15	
[11]	147709189 (203 kb upstream)	147976294 (25 kb downstream)	Dup	1								clinical testing for seizures (lab)	CNV 2H_0 4C_0.15x3 5A_0.15	VUS (0.6)
[11] #2	147796927 (115 kb upstream)	148144967 (194 kb downstream)	Dup het	1				De novo				clinical testing (lab)	CNV 2H_0 4C_0.15x3 5A_0.15	VUS (0.6)
[25]	147894723 (17 kb upstream)	147979356 (28 kb downstream)	Dup	1	29	hg18: 1q44[245,377,586-245,556,179]x3 (from unaffected father), 4p15.31[22,897,572-23,066,434]x1		De novo			Normal mRNA level (leukocyte qRT-PCR)	Myoclonic seizures	CNV 2H_0 4C_0.15x3 5A_0.15	VUS (0.6)
[26]	147912003 (c.-177)	147912051 (c.-129)	Dup	N/A (0.2-0.3% X chromosomes in study)						Present in multiple unaffected males		Finnish population samples	CNV 2I_0 4O_-1	BEN (-1)

1. Quan, F.; Zonana, J.; Gunter, K.; Peterson, K.L.; Magenis, R.E.; Popovich, B.W. An atypical case of fragile X syndrome caused by a deletion that includes the FMR1 gene. *Am J Hum Genet* **1995**, *56*, 1042-1051.
2. Parvari, R.; Mumm, S.; Galil, A.; Manor, E.; Bar-David, Y.; Carmi, R. Deletion of 8.5 Mb, including the FMR1 gene, in a male with the fragile X syndrome phenotype and overgrowth. *Am J Med Genet* **1999**, *83*, 302-307, doi:10.1002/(sici)1096-8628(19990402)83:4<302::aid-ajmg13>3.0.co;2-p.
3. Myers, K.A.; van 't Hof, F.N.G.; Sadleir, L.G.; Legault, G.; Simard-Tremblay, E.; Amor, D.J.; Scheffer, I.E. Fragile Females: Case Series of Epilepsy in Girls With FMR1 Disruption. *Pediatrics* **2019**, *144*, doi:10.1542/peds.2019-0599.

4. Coffee, B.; Ikeda, M.; Budimirovic, D.B.; Hjelm, L.N.; Kaufmann, W.E.; Warren, S.T. Mosaic FMR1 deletion causes fragile X syndrome and can lead to molecular misdiagnosis: a case report and review of the literature. *Am J Med Genet A* **2008**, *146a*, 1358-1367, doi:10.1002/ajmg.a.32261.
5. Wöhrle, D.; Kotzot, D.; Hirst, M.C.; Manca, A.; Korn, B.; Schmidt, A.; Barbi, G.; Rott, H.D.; Poustka, A.; Davies, K.E.; et al. A microdeletion of less than 250 kb, including the proximal part of the FMR-I gene and the fragile-X site, in a male with the clinical phenotype of fragile-X syndrome. *Am J Hum Genet* **1992**, *51*, 299-306.
6. Jiraanont, P.; Hagerman, R.J.; Neri, G.; Zollino, M.; Murdolo, M.; Tassone, F. Germinal mosaicism for a deletion of the FMR1 gene leading to fragile X syndrome. *Eur J Med Genet* **2016**, *59*, 459-462, doi:10.1016/j.ejmg.2016.08.009.
7. Trottier, Y.; Imbert, G.; Poustka, A.; Fryns, J.P.; Mandel, J.L. Male with typical fragile X phenotype is deleted for part of the FMR1 gene and for about 100 kb of upstream region. *Am J Med Genet* **1994**, *51*, 454-457, doi:10.1002/ajmg.1320510431.
8. Hirst, M.; Grewal, P.; Flannery, A.; Slatter, R.; Maher, E.; Barton, D.; Fryns, J.P.; Davies, K. Two new cases of FMR1 deletion associated with mental impairment. *Am J Hum Genet* **1995**, *56*, 67-74.
9. Prior, T.W.; Papp, A.C.; Snyder, P.J.; Sedra, M.S.; Guida, M.; Enrile, B.G. Germline mosaicism at the fragile X locus. *Am J Med Genet* **1995**, *55*, 384-386, doi:10.1002/ajmg.1320550327.
10. Griswold, A.J.; Ma, D.; Cukier, H.N.; Nations, L.D.; Schmidt, M.A.; Chung, R.H.; Jaworski, J.M.; Salyakina, D.; Konidari, I.; Whitehead, P.L.; et al. Evaluation of copy number variations reveals novel candidate genes in autism spectrum disorder-associated pathways. *Hum Mol Genet* **2012**, *21*, 3513-3523, doi:10.1093/hmg/dds164.
11. Nagamani, S.C.; Erez, A.; Probst, F.J.; Bader, P.; Evans, P.; Baker, L.A.; Fang, P.; Bertin, T.; Hixson, P.; Stankiewicz, P.; et al. Small genomic rearrangements involving FMR1 support the importance of its gene dosage for normal neurocognitive function. *Neurogenetics* **2012**, *13*, 333-339, doi:10.1007/s10048-012-0340-y.
12. Gu, Y.; Lugenbeel, K.A.; Vockley, J.G.; Grody, W.W.; Nelson, D.L. A de novo deletion in FMR1 in a patient with developmental delay. *Hum Mol Genet* **1994**, *3*, 1705-1706, doi:10.1093/hmg/3.9.1705.
13. Luo, S.; Huang, W.; Xia, Q.; Du, Q.; Wu, L.; Duan, R. Mutational analyses of the FMR1 gene in Chinese pediatric population of fragile x suspects: low tolerance for point mutation. *J Child Neurol* **2015**, *30*, 803-806, doi:10.1177/0883073814538508.
14. Luo, S.; Huang, W.; Xia, Q.; Xia, Y.; Du, Q.; Wu, L.; Duan, R. Cryptic FMR1 mosaic deletion in a phenotypically normal mother of a boy with fragile X syndrome: case report. *BMC Med Genet* **2014**, *15*, 125, doi:10.1186/s12881-014-0125-2.
15. Meijer, H.; de Graaff, E.; Merckx, D.M.; Jongbloed, R.J.; de Die-Smulders, C.E.; Engelen, J.J.; Fryns, J.P.; Curfs, P.M.; Oostra, B.A. A deletion of 1.6 kb proximal to the CGG repeat of the FMR1 gene causes the clinical phenotype of the fragile X syndrome. *Hum Mol Genet* **1994**, *3*, 615-620, doi:10.1093/hmg/3.4.615.
16. Hammond, L.S.; Macias, M.M.; Tarleton, J.C.; Shashidhar Pai, G. Fragile X syndrome and deletions in FMR1: new case and review of the literature. *Am J Med Genet* **1997**, *72*, 430-434.

17. Collins, S.C.; Coffee, B.; Benke, P.J.; Berry-Kravis, E.; Gilbert, F.; Oostra, B.; Halley, D.; Zwick, M.E.; Cutler, D.J.; Warren, S.T. Array-based FMR1 sequencing and deletion analysis in patients with a fragile X syndrome-like phenotype. *PLoS One* **2010**, *5*, e9476, doi:10.1371/journal.pone.0009476.
18. Tabolacci, E.; Pietrobono, R.; Maneri, G.; Remondini, L.; Nobile, V.; Della Monica, M.; Pomponi, M.G.; Genuardi, M.; Neri, G.; Chiurazzi, P. Reversion to Normal of FMR1 Expanded Alleles: A Rare Event in Two Independent Fragile X Syndrome Families. *Genes (Basel)* **2020**, *11*, doi:10.3390/genes11030248.
19. Hung, C.C.; Lee, C.N.; Wang, Y.C.; Chen, C.L.; Lin, T.K.; Su, Y.N.; Lin, M.W.; Kang, J.; Tai, Y.Y.; Hsu, W.W.; et al. Fragile X syndrome carrier screening in pregnant women in Chinese Han population. *Sci Rep* **2019**, *9*, 15456, doi:10.1038/s41598-019-51726-4.
20. Grønskov, K.; Hjalgrim, H.; Bjerager, M.O.; Brøndum-Nielsen, K. Deletion of all CGG repeats plus flanking sequences in FMR1 does not abolish gene expression. *Am J Hum Genet* **1997**, *61*, 961-967, doi:10.1086/514872.
21. Erbs, E.; Fenger-Grøn, J.; Jacobsen, C.M.; Lildballe, D.L.; Rasmussen, M. Spontaneous rescue of a FMR1 repeat expansion and review of deletions in the FMR1 non-coding region. *Eur J Med Genet* **2021**, *64*, 104244, doi:10.1016/j.ejmg.2021.104244.
22. Redin, C.; Gérard, B.; Lauer, J.; Herenger, Y.; Muller, J.; Quartier, A.; Masurel-Paulet, A.; Willems, M.; Lesca, G.; El-Chehadeh, S.; et al. Efficient strategy for the molecular diagnosis of intellectual disability using targeted high-throughput sequencing. *J Med Genet* **2014**, *51*, 724-736, doi:10.1136/jmedgenet-2014-102554.
23. Quartier, A.; Poquet, H.; Gilbert-Dussardier, B.; Rossi, M.; Casteleyn, A.S.; Portes, V.D.; Feger, C.; Nourisson, E.; Kuentz, P.; Redin, C.; et al. Intragenic FMR1 disease-causing variants: a significant mutational mechanism leading to Fragile-X syndrome. *Eur J Hum Genet* **2017**, *25*, 423-431, doi:10.1038/ejhg.2016.204.
24. Viveiros, M.T.; Santos, M.D.; Dos Santos, J.M.; Viveiros, D.M.; Cavalcante, M.R.; Caldas, A.J.; Pimentel, M.M. Screening for fragile X syndrome in males from specialized institutions in the northeast region of Brazil. *Genet Mol Res* **2015**, *14*, 6897-6905, doi:10.4238/2015.June.18.32.
25. Vengoechea, J.; Parikh, A.S.; Zhang, S.; Tassone, F. De novo microduplication of the FMR1 gene in a patient with developmental delay, epilepsy and hyperactivity. *Eur J Hum Genet* **2012**, *20*, 1197-1200, doi:10.1038/ejhg.2012.78.
26. Mononen, T.; von Koskull, H.; Airaksinen, R.L.; Juvonen, V. A novel duplication in the FMR1 gene: implications for molecular analysis in fragile X syndrome and repeat instability. *Clin Genet* **2007**, *72*, 528-531, doi:10.1111/j.1399-0004.2007.00903.x.
