# Supplementary material for: Beyond Trinucleotide Repeat Expansion in Fragile X Syndrome: Rare Coding and Noncoding Variants in FMR1 and Associated Phenotypes
Source: Genes (Basel). 2021 Oct 22;12(11):1669. doi: 10.3390/genes12111669 (PMC8623550; doi:10.3390/genes12111669)
Supplement: Supplementary file 1 [file genes-12-01669-s001.zip › Supplementary Table S3.pdf]

Supplementary Table S3. All published coding region small variants with details of information used to apply pathogenicity criteria.

Ref.	Location		# affected with variant, sex	CGG repeats	Other variants in pt.	Inheritance in proband	Segregation	Patient and/or functional data	ACMG criteria [1]	Conclusion
	c.	p.								
[2]	c.80C>A	p.S27*	1M, 1F	29		Mat (affected)		FMRP absent	PVS1 PS3 PM2 PP1	PATH
[3]	c.188_193del	p.(D63_E64del)	1M	nl		De novo confirmed			PS2 PM2 PM4 PP3	LPATH
[4]	c.229delT	p.(C77Afs*5)	1M	nl	Maternal PATH 308 kb del involving *PTCHD1-AS* (autism)	De novo confirmed			PVS1 PS2 PM2 BP5	PATH
[5] #1	c.373delA[a]	p.(T125Lfs*35)	1M	nl		De novo		mRNA + (RT-PCR), FMRP absent (lymphoblastoid cell line)	PVS1 PS3 PM6	PATH
[6], [7][b]	c.377T>C	p.(F126S)	1M		*KDM5C* I1349M (maternal), *ALG13* H771R (maternal)	De novo			PS2 PM6 PP3	LPATH
[8], [9]	c.413G>A	p.R138Q	1M, 1F	nl		Maternal (affected, learning disability/anxiety)	MGF and father (not tested) also had learning problems	AMPA signaling and RNA binding unchanged (transduced mouse neurons); deficient presynaptic function (Drosophila)	PS3 PP1 PP4; 7 hemizygotes in gnomAD v3 exomes (rs200163413)	LPATH
[10]	c.413G>A	p.R138Q	1M	24	(1st cousin consanguinity)	Maternal	Present in sister (no details)	Classic FXS presentation		
[11]	c.413G>A	p.R138Q	N/A					ID/ASD-like features (knock-in mouse model)		
[12]	c.413G>A	p.R138Q	1F	29, 37		Absent in mother; healthy father not tested				
[13,14]	c.677G>A	p.(R226K)	1M		7 variants, no diagnosis				PM2 PP3	VUS
[13]	c.767A>G	p.(D256G)	1M		5 variants, no diagnosis				PM2 PP3	VUS

[15]	c.797G>A	p.(G266E)	1M	23		Maternal (unaffected)	Absent in 3 unaffected brothers	Deficient RNA binding, AMPA trafficking (transduced mouse neurons)	PS3 PM2 PP3	LPATH
[16], [17]	c.911T>A	p.(I304N)	1M	nl	Known PHKA2 deficiency (glycogen storage disease IXa1)	De novo confirmed		mRNA present (RT-PCR), deficient association with polyribosomes (lymphoblastoid cell line)	PS2 PS3 PM2 PP3	PATH
[18] #1	c.1021-1028delinsTATTGG	p.N341Yfs*7	2M	nl		Mother not tested; had epilepsy	Present in unaffected sister, absent in 2 unaffected brothers	mRNA +, FMRP absent (lymphoblastoid cell line)	PVS1 PS3 PP1	PATH
[19]	c.1325G>A	p.(R442Q)	2M + mother, grandmother		11 kb paternal deletion of part of *MYH7*	Maternal (affected)	Present in 2 affected brothers and mildly affected mother	FMRP nl in hair roots (4 family members); abnormal protein localization (transfected HEK cells)	PS3 PM2 PP1 PP3	LPATH
[20]	c.1444G>A	p.(G482S)	1M						PM2 BP4	VUS
[21]	c.1550C>T[c]	p.(P517L)	1M	36		?			PM2	VUS
[20]	c.1601G>A	p.(R534H)	2M (unrelated), 1F			Maternal (unaffected) in both families			PM2	VUS
[22]	c.1610dup	p.(G538Rfs*24)	1M	41		?		mRNA 60% decreased and FMRP >90% decreased (lymphocyte cell line) Truncated protein abnormal localization and function (transfected HEK cells, Drosophila)	PVS1 PS3 PM2	PATH
[23]	c.1618G>A	p.(G540E)	1M	nl					PM2 PP3	VUS
[24] TN351	c.1637G>A	p.(R546H)	1M	nl	*FMR1* c.990+14C>T (see Table 4)	?			PM2	VUS

[a] New BglII restriction site.
[b] All references in literature are to a variant identified in the DDD study that was not listed in that publication but corresponds to DECIPHER patient #259197.
[c] Reported as NM_001185081.1:c.1216C>T (p.(Q406*)) on an alternate reading frame from the reference transcript.

1. Richards, S.; Aziz, N.; Bale, S.; Bick, D.; Das, S.; Gastier-Foster, J.; Grody, W.W.; Hegde, M.; Lyon, E.; Spector, E.; et al. Standards and guidelines for the interpretation of sequence variants: a joint consensus recommendation of the American College of Medical Genetics and Genomics and the Association for Molecular Pathology. *Genet Med* **2015**, *17*, 405-424, doi:10.1038/gim.2015.30.
2. Grønskov, K.; Brøndum-Nielsen, K.; Dedic, A.; Hjalgrim, H. A nonsense mutation in FMR1 causing fragile X syndrome. *Eur J Hum Genet* **2011**, *19*, 489-491, doi:10.1038/ejhg.2010.223.
3. Maddirevula, S.; Alsaif, H.S.; Ibrahim, N.; Alkuraya, F.S. A de novo mutation in FMR1 in a patient with intellectual disability. *Eur J Med Genet* **2020**, *63*, 103763, doi:10.1016/j.ejmg.2019.103763.
4. Saldarriaga, W.; Payán-Gómez, C.; González-Teshima, L.Y.; Rosa, L.; Tassone, F.; Hagerman, R.J. Double Genetic Hit: Fragile X Syndrome and Partial Deletion of Protein Patched Homolog 1 Antisense as Cause of Severe Autism Spectrum Disorder. *J Dev Behav Pediatr* **2020**, *41*, 724-728, doi:10.1097/dbp.0000000000000850.
5. Lugenbeel, K.A.; Peier, A.M.; Carson, N.L.; Chudley, A.E.; Nelson, D.L. Intragenic loss of function mutations demonstrate the primary role of FMR1 in fragile X syndrome. *Nat Genet* **1995**, *10*, 483-485, doi:10.1038/ng0895-483.
6. Wright, C.F.; Fitzgerald, T.W.; Jones, W.D.; Clayton, S.; McRae, J.F.; van Kogelenberg, M.; King, D.A.; Ambridge, K.; Barrett, D.M.; Bayzetinova, T.; et al. Genetic diagnosis of developmental disorders in the DDD study: a scalable analysis of genome-wide research data. *Lancet* **2015**, *385*, 1305-1314, doi:10.1016/S0140-6736(14)61705-0.
7. Quartier, A.; Poquet, H.; Gilbert-Dussardier, B.; Rossi, M.; Casteleyn, A.S.; Portes, V.D.; Feger, C.; Nourisson, E.; Kuentz, P.; Redin, C.; et al. Intragenic FMR1 disease-causing variants: a significant mutational mechanism leading to Fragile-X syndrome. *Eur J Hum Genet* **2017**, *25*, 423-431, doi:10.1038/ejhg.2016.204.
8. Collins, S.C.; Bray, S.M.; Suhl, J.A.; Cutler, D.J.; Coffee, B.; Zwick, M.E.; Warren, S.T. Identification of novel FMR1 variants by massively parallel sequencing in developmentally delayed males. *Am J Med Genet A* **2010**, *152a*, 2512-2520, doi:10.1002/ajmg.a.33626.
9. Myrick, L.K.; Deng, P.Y.; Hashimoto, H.; Oh, Y.M.; Cho, Y.; Poidevin, M.J.; Suhl, J.A.; Visootsak, J.; Cavalli, V.; Jin, P.; et al. Independent role for presynaptic FMRP revealed by an FMR1 missense mutation associated with intellectual disability and seizures. *Proc Natl Acad Sci U S A* **2015**, *112*, 949-956, doi:10.1073/pnas.1423094112.
10. Sitzmann, A.F.; Hagelstrom, R.T.; Tassone, F.; Hagerman, R.J.; Butler, M.G. Rare FMR1 gene mutations causing fragile X syndrome: A review. *Am J Med Genet A* **2018**, *176*, 11-18, doi:10.1002/ajmg.a.38504.

11. Prieto, M.; Folci, A.; Poupon, G.; Schiavi, S.; Buzzelli, V.; Pronot, M.; François, U.; Pousinha, P.; Lattuada, N.; Abelanet, S.; et al. Missense mutation of Fmr1 results in impaired AMPAR-mediated plasticity and socio-cognitive deficits in mice. *Nat Commun* **2021**, *12*, 1557, doi:10.1038/s41467-021-21820-1.
12. Diaz, J.; Scheiner, C.; Leon, E. Presentation of a recurrent FMR1 missense mutation (R138Q) in an affected female. *Translational Science of Rare Diseases* **2018**, *3*, 139-144, doi:10.3233/TRD-180028.
13. Grozeva, D.; Carss, K.; Spasic-Boskovic, O.; Tejada, M.I.; Gecz, J.; Shaw, M.; Corbett, M.; Haan, E.; Thompson, E.; Friend, K.; et al. Targeted Next-Generation Sequencing Analysis of 1,000 Individuals with Intellectual Disability. *Hum Mutat* **2015**, *36*, 1197-1204, doi:10.1002/humu.22901.
14. Carion, N.; Briand, A.; Cuisset, L.; Pacot, L.; Afenjar, A.; Bienvenu, T. Loss of the KH1 domain of FMR1 in humans due to a synonymous variant causes global developmental retardation. *Gene* **2020**, *753*, 144793, doi:10.1016/j.gene.2020.144793.
15. Myrick, L.K.; Nakamoto-Kinoshita, M.; Lindor, N.M.; Kirmani, S.; Cheng, X.; Warren, S.T. Fragile X syndrome due to a missense mutation. *Eur J Hum Genet* **2014**, *22*, 1185-1189, doi:10.1038/ejhg.2013.311.
16. De Boulle, K.; Verkerk, A.J.; Reyniers, E.; Vits, L.; Hendrickx, J.; Van Roy, B.; Van den Bos, F.; de Graaff, E.; Oostra, B.A.; Willems, P.J. A point mutation in the FMR-1 gene associated with fragile X mental retardation. *Nat Genet* **1993**, *3*, 31-35, doi:10.1038/ng0193-31.
17. Feng, Y.; Absher, D.; Eberhart, D.E.; Brown, V.; Malter, H.E.; Warren, S.T. FMRP associates with polyribosomes as an mRNP, and the I304N mutation of severe fragile X syndrome abolishes this association. *Mol Cell* **1997**, *1*, 109-118, doi:10.1016/s1097-2765(00)80012-x.
18. Carroll, R.; Shaw, M.; Arvio, M.; Gardner, A.; Kumar, R.; Hodgson, B.; Heron, S.; McKenzie, F.; Järvelä, I.; Gecz, J. Two novel intragenic variants in the FMR1 gene in patients with suspect clinical diagnosis of Fragile X syndrome and no CGG repeat expansion. *Eur J Med Genet* **2020**, *63*, 104010, doi:10.1016/j.ejmg.2020.104010.
19. Zeidler, S.; Severijnen, L.A.; de Boer, H.; van der Toorn, E.C.; Ruivenkamp, C.A.L.; Bijlsma, E.K.; Willemsen, R. A missense variant in the nuclear export signal of the FMR1 gene causes intellectual disability. *Gene* **2021**, *768*, 145298, doi:10.1016/j.gene.2020.145298.
20. Handt, M.; Epplen, A.; Hoffjan, S.; Mese, K.; Epplen, J.T.; Dekomien, G. Point mutation frequency in the FMR1 gene as revealed by fragile X syndrome screening. *Mol Cell Probes* **2014**, *28*, 279-283, doi:10.1016/j.mcp.2014.08.003.
21. Park, K.M.; Jun, K.R.; Lee, H.J.; Park, S.; Kim, S.E. Unilateral diffuse white matter involvement in a patient with atypical FMR1 mutation. *Clin Neurol Neurosurg* **2020**, *197*, 106182, doi:10.1016/j.clineuro.2020.106182.
22. Okray, Z.; de Esch, C.E.; Van Esch, H.; Devriendt, K.; Claeys, A.; Yan, J.; Verbeeck, J.; Froyen, G.; Willemsen, R.; de Vrij, F.M.; et al. A novel fragile X syndrome mutation reveals a conserved role for the carboxy-terminus in FMRP localization and function. *EMBO Mol Med* **2015**, *7*, 423-437, doi:10.15252/emmm.201404576.

23. Hu, H.; Haas, S.A.; Chelly, J.; Van Esch, H.; Raynaud, M.; de Brouwer, A.P.; Weinert, S.; Froyen, G.; Frints, S.G.; Laumonnier, F.; et al. X-exome sequencing of 405 unresolved families identifies seven novel intellectual disability genes. *Mol Psychiatry* **2016**, *21*, 133-148, doi:10.1038/mp.2014.193.
24. Wang, Y.C.; Lin, M.L.; Lin, S.J.; Li, Y.C.; Li, S.Y. Novel point mutation within intron 10 of FMR-1 gene causing fragile X syndrome. *Hum Mutat* **1997**, *10*, 393-399, doi:10.1002/(sici)1098-1004(1997)10:5<393::Aid-humu10>3.0.Co;2-v.
