# Supplementary material for: Beyond Trinucleotide Repeat Expansion in Fragile X Syndrome: Rare Coding and Noncoding Variants in FMR1 and Associated Phenotypes
Source: Genes (Basel). 2021 Oct 22;12(11):1669. doi: 10.3390/genes12111669 (PMC8623550; doi:10.3390/genes12111669)
Supplement: Supplementary file 1 [file genes-12-01669-s001.zip › Supplementary Table S5.pdf]

Supplementary Table 5. All published noncoding small variants with details of information used to apply pathogenicity criteria.

Ref.	Location		# affected with variant, sex	CGG repeats	Other testing done, variants	Inheritance in proband	Patient and/or functional data	ACMG criteria	Conclusion
	DNA	r./p.							
[1]	g.147909332G>A	N/A (2.5 kb upstream of r.1, loss of EcoRI site)	1F	23, 32		Paternal (unaffected)	(1 het in gnomAD)	BS2	VUS
[2] #2	g.147911760_2dup(GGC) (promoter ~-154)		1M	31	FMR1 only		Absent in unaffected sister<br>Normal FMRP (lymphocytes), normal gel shift and reporter assays *in vitro*	BS1 BS2 BS3	BEN
[3]	g.147911760_2dup(GGC) (promoter ~-154)		8 samples (male DD cohort)	nl	FMR1 only		(gnomAD AF 2.5% with many hemizygotes, rs200904100)		
[4]	g.147911767C>G (promoter ~-149)		1M	31	FMR1 only		Present in unaffected MGF (and mother, mat aunt, and sister)	BS2 BS3	BEN
[2] #1	g.147911767C>G (promoter ~-149)		1M	29	AFF2 repeats and FMR1		Normal gel shift and gene reporter assays		
[3]	"c.-332G>C" (promoter)		1M (male DD cohort)	nl	FMR1 only		Reduced reporter expression to 5.9% of WT (10 hemizygotes in gnomAD, rs922007219)	BS1 BS2 PS3	VUS
[3]	"c.-293T>C" (promoter)		1M (male DD cohort)	nl	FMR1 only		Reduced reporter expression to 29.2% of WT (7 hemizygotes in gnomAD, rs1222840333)	BS1 BS2 PS3	VUS
[3]	c.-254A>G		1M (male DD cohort)	nl	FMR1 only		Reduced reporter expression to 36.2% of WT (8 hemizygotes in gnomAD, rs1217601043)	BS1 BS2 PS3	VUS
[5]	CGG repeat 8 CGG>CCG (c.-107G>C)	N/A (new EagI site)	N/A (healthy female, FHx ID)	nl	FMR1 repeat region only		Present in 2 unaffected males, opposite side of family from members with ID	BS2 BS4	BEN

[6]	CGG repeat 26 of 31 CGG>CCG	N/A (new EagI site)	1M	31	FMR1 partial sequencing only	Maternal (unaffected)	Normal % of FMRP+ lymphocytes, but statistically significant decrease to 76% normal FMRP level in lymphoblastoid cell line	None	VUS
[7]	c.-4_+1delGAAGA	p.(M1=), no start loss because c.-9_-5 is also GAAGA (amplification failure in PCR assay)	1M	nl	FMR1 only	Maternal (98% FMRP+ lymphocytes)	Normal FMRP level in lymphoblastoid cell line; 80% lymphocytes FMRP+	BS3	VUS
[8]	c.18G>T	p.(V6=)	2M (unrelated)	nl	FMR1 only	Maternal (unaffected) in 1	Normal intron 1 splicing in lymphoblastoid cell line (1 patient), normal FMRP Western blot in lymphoblastoid cell line (1 patient)		
[9]	c.18G>T	p.(V6=)	4/508 male ID/DD cohort					BS1 BS2 BS3	BEN
[3]	c.18G>T	p.(V6=)	13 samples, 19 controls				Observed in multiple unaffected controls		
[8]	c.51+88_89del	(intron 1)	1M (clinical FXS testing)	nl	FMR1 only		Absent in affected brother	BS4	VUS
[10]	c.51+423G>C	(New BssHI site on Southern)	1M	42	FMR1 partial sequencing				VUS
[11]	c.51+730A>G	(New EcoRI site on Southern)	1M (clinical lab testing)	nl	FMR1 only	Maternal (unaffected)	Present in 1 brother, ? phenotype		VUS
[12]	c.52-47A>G		1M (ID, 2 FXS features)		FMR1 sequencing-by-hybridization		(221 hemizygotes in gnomAD, rs80358323)	BS1 BS2	BEN
[13]	c.52-1_52delinsTA	2 abnormal transcripts	1M + mother	nl	FMR1 partial sequencing	Maternal (affected)	No normal transcripts; 2 abnormal transcripts with skipped exon 2 and skipped exons 2-3; FMRP absent in lymphoblastoid cell line	PVS1 PS3 PP1	PATH

[12]	c.105-179G>T		1M (ID, 2 FXS features)		FMR1 sequencing-by-hybridization		(305 hemizygotes in gnomAD, rs80358324)	BS1 BS2	BEN
[3]	c.105-8A>C		6/963 samples (male DD cohort)	nl	FMR1 only		(103 hemizygotes in gnomAD, rs183745963)	BS1 BS2	BEN
[14] #3	c.420-8A>G	r.419_420ins420-7_420-1 (p.(M140Ifs*3))	1M (ID cohort)	nl	451-gene ID exon panel	Maternal (unaffected, no XCI skewing)	Cryptic splice acceptor leading to retention of 7 nt from intron 5 (blood)	PS3 PM2	LPATH
[15]	c.513+27T>C	No change found (intron 6)	1 sample (ID, 1 FXS feature cohort)	nl	FMR1 only		No change in exon 6-7 junction on RT-PCR product sequencing	BS3	VUS
[3]	*c.630+438A>C*		1M (DD cohort)	nl	FMR1 only				VUS
[3]	*c.631-840G>A*		1M (DD cohort)	nl	FMR1 only				VUS
[16,17]	c.801G>A (IVS8-1)	r.631_801del (p.(S211_G267del)); exon 8 skipping	1M (ID cohort)		56-gene ID panel	De novo with maternity/paternity confirmed	No FHx DD (parental first cousin consanguinity)<br>Exon 8 skipping with no normal RT-PCR product; rat model with deletion of exon 8 is affected	PS2 PS3 PM2	PATH
[18]	c.879A>C (IVS9-2)	p.(V293=); reported abnormal splicing intron 9	1F (autism/ ID cohort)	nl	FMR1 partial (SSCP with subclone sequencing)		Inclusion of intron 9 sequence in 23/36 subclones from peripheral blood cDNA	BS2	VUS
[15]	c.879A>C (IVS9-2)	p.(V293=); no splicing abnormality found	1M (ID, 1 FXS feature)	nl	FMR1 only	Maternal (unaffected)	No abnormal splice amplicons found; normal exon 9-10 junction in RT-PCR product sequence in blood; FMRP present in blood homogenate<br>(1 hemizygote in gnomAD, rs782013865)		
[3]	c.880+885A>G		1M (DD cohort)	nl	FMR1 only		(24 hemizygotes in gnomAD, rs781933453)	BS1 BS2	BEN

[19]	c.881-1G>T	? (exon 10 splice acceptor)	1M (clinical suspicion)		Genome sequencing	Maternal (1:99 skewed XCI, unaffected)		PVS1 PM2	LPATH
[14]	c.990+1G>A	r.881_990del (p.(K295Nfs*11)) (exon 10 skipping)	1M (ID cohort)	nl	451-gene ID exon panel	De novo	Exon 10 skipping in blood	PVS1 PS3 PM2 PM6	PATH
[3]	*c.990+4T>C*	? (intron 10)	1M (DD cohort)	nl	FMR1 only			PM2	VUS
[20]	c.990+14C>T	r.881_990del (p.(K295Nfs*11)) (exon 10 skipping)	3 unrelated males (ID, FXS feature cohort)	nl	SSCP exons 1-10 and 15 with sequencing; c.1637G>A in one patient		Exon 10 skipping on peripheral blood RT-PCR product sequencing in 2 probands (TN-183, TN-351)	BA1 BS2	BEN
[21]	c.990+14C>T		81 control individuals				Observed in many controls from general population		
[9]	c.990+14C>T		45/508 in ID/DD cohort						
[22]	c.990+14C>T		7M/4F among 88 patients with ASD				Statistically significant (p=0.0123) higher frequency in ASD patients vs controls Stably inherited in unaffected family members		
[23]	c.990+14C>T						No significant transmission disequilibrium (p=0.26) Allele frequency 65% (22/34) in East Asian controls; concluded that previously observed association with ASD was false positive due to population stratification (gnomAD AF >10%, rs25714)		
[9]	c.1189-39A>G		1M (ID/DD cohort)		FMR1 melting and		(2 hemizygotes in gnomAD, rs781962133)	BS2	VUS

					sequencing only			
[3]	c.1472-521C>G		1M (DD cohort)		FMR1 only	(1 hemizygote in gnomAD, rs1557181482)	BS2	VUS
[3]	c.*23T>C		1M (DD cohort)		FMR1 only			VUS
[9]	c.*60G>C		1M (ID/DD cohort)		FMR1 melting and sequencing only	(46 hemizygotes in gnomAD, rs782402226)	BS1 BS2	BEN
[9]	c.*68T>C		1M (ID/DD cohort)		FMR1 melting and sequencing only	(2 hemizygotes in gnomAD, rs781983693)	BS2	VUS
[15]	c.*312_313dupT		1 (ID, 1 FXS feature cohort)	nl	FMR1 only		PM2	VUS
[24], [3]	c.*746T>C		2M (proband and half-brother from DD cohort)	nl	FMR1 only	Faster mRNA decay with FMRP level 80% of normal in lymphoblastoid cell line; variant sufficient and necessary to decrease reporter gene expression *in vitro;* loss of metabotropic glutamate receptor-stimulated upregulation of reporter expression in transfected mouse neurons (72 hemizygotes in gnomAD, rs183130936)	PS3 PP1 BS1 BS2	VUS
[15]	c.*760C>A		1 (ID, 1 FXS feature cohort)	nl	FMR1 only	(1 hemizygote in gnomAD, rs184987604)	BS2	VUS
[3]	c.*1867G>A		12/963 samples (DD cohort)	nl	FMR1 only	(466 hemizygotes in gnomAD, rs148216485)	BS1 BS2	BEN
[3]	c.*2035C>T		3/963 samples (DD cohort)	nl	FMR1 only	(39 hemizygotes in gnomAD, rs140123351)	BS1 BS2	BEN

1. Liang, S.; Bass, H.N.; Gao, H.; Astbury, C.; Jamehdor, M.R.; Qu, Y. A pseudo-full mutation identified in fragile X assay reveals a novel base change abolishing an EcoRI restriction site. *J Mol Diagn* **2008**, *10*, 469-474, doi:10.2353/jmoldx.2008.080059.
2. Milà, M.; Castellví-Bel, S.; Sánchez, A.; Barceló, A.; Badenas, C.; Mallolas, J.; Estivill, X. Rare variants in the promoter of the fragile X syndrome gene (FMR1). *Mol Cell Probes* **2000**, *14*, 115-119, doi:10.1006/mcpr.2000.0293.
3. Collins, S.C.; Bray, S.M.; Suhl, J.A.; Cutler, D.J.; Coffee, B.; Zwick, M.E.; Warren, S.T. Identification of novel FMR1 variants by massively parallel sequencing in developmentally delayed males. *Am J Med Genet A* **2010**, *152a*, 2512-2520, doi:10.1002/ajmg.a.33626.
4. Grasso, M.; Cecconi, M.; Boni, S.; Forzano, F.; Barbaresi, M.; Memo, L.; Perroni, L.; Faravelli, F.; Di Maria, E. The -413C > G substitution in the promoter of the FMR1 gene is not associated with the fragile X syndrome phenotype. *Mol Cell Probes* **2010**, *24*, 107-109, doi:10.1016/j.mcp.2009.10.006.
5. Cecconi, M.; Forzano, F.; Rinaldi, R.; Cappellacci, S.; Grammatico, P.; Faravelli, F.; Dagna Bricarelli, F.; Di Maria, E.; Grasso, M. A single nucleotide variant in the FMR1 CGG repeat results in a "Pseudodeletion" and is not associated with the fragile X syndrome phenotype. *J Mol Diagn* **2008**, *10*, 272-275, doi:10.2353/jmoldx.2008.070163.
6. Tarleton, J.; Kenneson, A.; Taylor, A.K.; Crandall, K.; Fletcher, R.; Casey, R.; Hart, P.S.; Hatton, D.; Fisch, G.; Warren, S.T. A single base alteration in the CGG repeat region of FMR1: possible effects on gene expression and phenotype. *J Med Genet* **2002**, *39*, 196-200, doi:10.1136/jmg.39.3.196.
7. Hegde, M.R.; Chong, B.; Fawkner, M.; Lambiris, N.; Peters, H.; Kenneson, A.; Warren, S.T.; Love, D.R.; McGaughran, J. Microdeletion in the FMR-1 gene: an apparent null allele using routine clinical PCR amplification. *J Med Genet* **2001**, *38*, 624-629, doi:10.1136/jmg.38.9.624.
8. Grønskov, K.; Hallberg, A.; Brøndum-Nielsen, K. Mutational analysis of the FMR1 gene in 118 mentally retarded males suspected of fragile X syndrome: absence of prevalent mutations. *Hum Genet* **1998**, *102*, 440-445, doi:10.1007/s004390050718.
9. Handt, M.; Epplen, A.; Hoffjan, S.; Mese, K.; Epplen, J.T.; Dekomien, G. Point mutation frequency in the FMR1 gene as revealed by fragile X syndrome screening. *Mol Cell Probes* **2014**, *28*, 279-283, doi:10.1016/j.mcp.2014.08.003.
10. Thyagarajan, B.; Bower, M.; Berger, M.; Jones, S.; Dolan, M.; Wang, X. A novel polymorphism in the FMR1 gene: implications for clinical testing of fragile X syndrome. *Arch Pathol Lab Med* **2008**, *132*, 95-98, doi:10.5858/2008-132-95-anpitf.
11. Daly, T.M.; Rafii, A.; Martin, R.A.; Zehnbauer, B.A. Novel polymorphism in the FMR1 gene resulting in a "pseudodeletion" of FMR1 in a commonly used fragile X assay. *J Mol Diagn* **2000**, *2*, 128-131, doi:10.1016/s1525-1578(10)60627-7.

12. Collins, S.C.; Coffee, B.; Benke, P.J.; Berry-Kravis, E.; Gilbert, F.; Oostra, B.; Halley, D.; Zwick, M.E.; Cutler, D.J.; Warren, S.T. Array-based FMR1 sequencing and deletion analysis in patients with a fragile X syndrome-like phenotype. *PLoS One* **2010**, *5*, e9476, doi:10.1371/journal.pone.0009476.
13. Lugenbeel, K.A.; Peier, A.M.; Carson, N.L.; Chudley, A.E.; Nelson, D.L. Intragenic loss of function mutations demonstrate the primary role of FMR1 in fragile X syndrome. *Nat Genet* **1995**, *10*, 483-485, doi:10.1038/ng0895-483.
14. Quartier, A.; Poquet, H.; Gilbert-Dussardier, B.; Rossi, M.; Casteleyn, A.S.; Portes, V.D.; Feger, C.; Nourisson, E.; Kuentz, P.; Redin, C.; et al. Intragenic FMR1 disease-causing variants: a significant mutational mechanism leading to Fragile-X syndrome. *Eur J Hum Genet* **2017**, *25*, 423-431, doi:10.1038/ejhg.2016.204.
15. Luo, S.; Huang, W.; Xia, Q.; Du, Q.; Wu, L.; Duan, R. Mutational analyses of the FMR1 gene in Chinese pediatric population of fragile x suspects: low tolerance for point mutation. *J Child Neurol* **2015**, *30*, 803-806, doi:10.1177/0883073814538508.
16. Golden, C.E.M.; Breen, M.S.; Koro, L.; Sonar, S.; Niblo, K.; Browne, A.; Burlant, N.; Di Marino, D.; De Rubeis, S.; Baxter, M.G.; et al. Deletion of the KH1 Domain of Fmr1 Leads to Transcriptional Alterations and Attentional Deficits in Rats. *Cereb Cortex* **2019**, *29*, 2228-2244, doi:10.1093/cercor/bhz029.
17. Carion, N.; Briand, A.; Cuisset, L.; Pacot, L.; Afenjar, A.; Bienvenu, T. Loss of the KH1 domain of FMR1 in humans due to a synonymous variant causes global developmental retardation. *Gene* **2020**, *753*, 144793, doi:10.1016/j.gene.2020.144793.
18. Shinahara, K.; Saijo, T.; Mori, K.; Kuroda, Y. Single-strand conformation polymorphism analysis of the FMR1 gene in autistic and mentally retarded children in Japan. *J Med Invest* **2004**, *51*, 52-58, doi:10.2152/jmi.51.52.
19. Carroll, R.; Shaw, M.; Arvio, M.; Gardner, A.; Kumar, R.; Hodgson, B.; Heron, S.; McKenzie, F.; Järvelä, I.; Gecz, J. Two novel intragenic variants in the FMR1 gene in patients with suspect clinical diagnosis of Fragile X syndrome and no CGG repeat expansion. *Eur J Med Genet* **2020**, *63*, 104010, doi:10.1016/j.ejmg.2020.104010.
20. Wang, Y.C.; Lin, M.L.; Lin, S.J.; Li, Y.C.; Li, S.Y. Novel point mutation within intron 10 of FMR-1 gene causing fragile X syndrome. *Hum Mutat* **1997**, *10*, 393-399, doi:10.1002/(sici)1098-1004(1997)10:5<393::Aid-humu10>3.0.Co;2-v.
21. Vincent, J.B.; Gurling, H.M. Point mutation in intron 10 of FMR1 is unlikely to be a cause of fragile X syndrome. *Hum Mutat* **1998**, *12*, 431-432, doi:10.1002/(sici)1098-1004(1998)12:6<431::Aid-humu10>3.0.Co;2-y.
22. Vincent, J.B.; Konecki, D.S.; Munstermann, E.; Bolton, P.; Poustka, A.; Poustka, F.; Gurling, H.M. Point mutation analysis of the FMR-1 gene in autism. *Mol Psychiatry* **1996**, *1*, 227-231.
23. Vincent, J.B.; Thevarkunnel, S.; Kolozsvari, D.; Paterson, A.D.; Roberts, W.; Scherer, S.W. Association and transmission analysis of the FMR1 IVS10 + 14C-T variant in autism. *Am J Med Genet B Neuropsychiatr Genet* **2004**, *125b*, 54-56, doi:10.1002/ajmg.b.20088.

24. Suhl, J.A.; Muddashetty, R.S.; Anderson, B.R.; Ifrim, M.F.; Visootsak, J.; Bassell, G.J.; Warren, S.T. A 3' untranslated region variant in FMR1 eliminates neuronal activity-dependent translation of FMRP by disrupting binding of the RNA-binding protein HuR. *Proc Natl Acad Sci U S A* **2015**, *112*, E6553-6561, doi:10.1073/pnas.1514260112.
